# Carboxypeptidase inhibitors from *Solanaceae* as a new subclass of pathogenesis related peptide aiming biotechnological targets for plant defense

**DOI:** 10.3389/fmolb.2023.1259026

**Published:** 2023-11-16

**Authors:** Geniana da Silva Gomes, Paula Corrêa Espósito, Maria Cristina Baracat-Pereira

**Affiliations:** Laboratory of Proteomics and Protein Biochemistry, Department of Biochemistry and Molecular Biology, Universidade Federal de Viçosa, Viçosa, Brazil

**Keywords:** *Solanaceae*, carboxypeptidase inhibitors, antimicrobial peptides, plant defense, characterization, biotechnology, pathogen

## Abstract

**Background:** Plant protease inhibitors play a crucial role in inhibiting proteases produced by phytopathogens and exhibiting inhibitory effects on nematodes, fungi, and insects, making them promising candidates for crop protection. Specifically, carboxypeptidase inhibitors, a subset of proteinase inhibitors, have been extensively studied in potato and tomato of *Solanaceae* plant family. However, further research is needed to fully understand the functions and biotechnological potential of those inhibitors in plants. This work aimed to *in silico* characterize carboxypeptidase inhibitors from *Solanaceae* as potential antimicrobial and defense agents focused on biotechnological targets.

**Methods:** The methodology employed involved search in UniProt, PDB, KNOTTIN, NCBI, and MEROPS databases for solanaceous carboxypeptidase inhibitors, phylogenetic relationships and conservation patterns analyzes using MEGA-X software and Clustal Omega/MView tools, physicochemical properties and antimicrobial potential prediction using ProtParam, ToxinPred, iAMPred, and APD3 tools, and structural features prediction using PSIPRED.

**Results and discussion:** A systematic literature search was conducted to identify relevant studies on *Solanaceae* carboxypeptidase inhibitors and their activities against pathogens. The selected studies were reviewed and the main findings compiled. The characterization of *Solanaceae* carboxypeptidase inhibitors proposed for the first time the global sequence consensus motif CXXXCXXXXDCXXXXXCXXC, shedding light on carboxypeptidase inhibitors distribution, sequence variability, and conservation patterns. Phylogenetic analysis showed evolutionary relationships within the *Solanaceae* family, particularly in *Capsicum*, *Nicotiana*, and *Solanum* genera. Physicochemical characteristics of those peptides indicated their similarity to antimicrobial peptides. Predicted secondary structures exhibited variations, suggesting a broad spectrum of action, and studies had been demonstrated their activities against various pathogens.

**Conclusion:** Carboxypeptidase inhibitors are being proposed here as a new subclass of PR-6 pathogenesis-related proteins, which will aid in a focused understanding of their functional roles in plant defense mechanisms. These findings confirm the *Solanaceae* carboxypeptidase inhibitors potential as defense agents and highlight opportunities for their biotechnological applications in pathogen control.

## 1 Introduction

Plants employ various defense mechanisms to protect themselves against pathogens. They possess inherent barriers such as the cuticle layer and lignin depositions, which serve as physical and chemical obstacles to pathogen invasion (Landa et al., 2002; Chassot et al., 2007; Sels et al., 2008). Additionally, to these preexisting defenses, plants can activate inducible defense mechanisms upon pathogen attack. These mechanisms include reinforcement of the cell wall, initiation of a hypersensitive response, generation of reactive oxygen species, accumulation of secondary metabolites, and production of pathogenesis-related proteins (PR-proteins), including antimicrobial peptides (AMPs) (Durrant and Dong, 2004; Truman et al., 2007; Sels et al., 2008). Antimicrobial peptides, also referred to as plant defense peptides, play a critical role in the innate immune response of plants against pathogens. Many plant AMPs are small, cationic, and amphipathic molecules that exhibit broad-spectrum antimicrobial activity. They are capable of directly interacting with the cell membranes of pathogens, causing damages and eventual cell death (García-Olmedo et al., 2001; Li et al., 2021; Mazurkiewicz-Pisarek et al., 2023). Also, AMPs function as protease inhibitors decreasing, for instance, the ability of pathogens to invade plants (Nair et al., 2022). By functioning as potent natural antimicrobials, these peptides contribute to the defense arsenal of plants and provide an effective line of defense against invading pathogens.

AMPs can be classified into various families of pathogenesis-related (PR) proteins. PR proteins are a group of proteins or peptides induced in plants upon pathogen attack, serving as a crucial part of their defense system. Some notable classes include thionins (PR-13), defensins (PR-12), lipid transfer proteins (PR-14), hevein-like peptides (PR-3, PR-4, PR-8, and PR-11), knottin-like peptides, and protease inhibitors (PR-6) (García-Olmedo et al., 2001; Sels et al., 2008; Kaur et al., 2022). Extensive research has demonstrated the antimicrobial properties of PR proteins, highlighting their role in plant defense against pathogens. Studies focused on *Solanaceae* plants, such as *Solanum tuberosum* (potato) and *Solanum lycopersicum* (tomato), have revealed the involvement of PR proteins in antimicrobial activity and defense responses against phytopathogenic bacteria, fungi, viruses, and insects. For instance, the overexpression of the antimicrobial potato Snakin-1 in transgenic *Citrange troyer* resulted in reduced disease symptoms caused by the bacterium *Xanthomonas citri* pv. *citri* (Conti et al., 2020). In tomato, PR protein expression genes are associated with defense against insects such as *Trialeurodes vaporariorum* and *Bemisia tabaci*, as well as viruses like *Tobacco mosaic* virus and *Cucumber mosaic* virus (Puthoff et al., 2010; Aseel et al., 2023). These findings underscore the significance of PR proteins in plant defense mechanisms, particularly in the context of *Solanaceae* plants.

Plant protease inhibitors (PPIs) classified within the PR-6 protein family have gained significant attention in various research studies due to their crucial roles in plant defense against pathogens. These peptides function by inhibiting the activity of proteases, which are enzymes produced by pathogens to degrade plant proteins and facilitate their invasion. By inhibiting these proteases, PPIs can disrupt essential physiological processes of pathogens, ultimately leading to their suppression or elimination (Cotabarren et al., 2020; Godbole and Kharat, 2022). Moreover, these protease inhibitors (PIs) have been found to exhibit a wide range of inhibitory effects, including the suppression of nematode infections and the inhibition of growth in various pathogenic fungi (Terras et al., 1993; Urwin et al., 1997; Joshi et al., 1998; Have et al., 2004). Additionally, PPIs have demonstrated the ability to inhibit insect development, resulting in a decrease in the larval growth stage, highlighting their potential as transgenic resistance factors (Shukle and Wu, 2003). Given the wide spectrum of action observed in plant PPIs, they could be promising candidates for biotechnological applications (Cotabarren et al., 2020). Their multifunctional properties, including antimicrobial and insecticidal activities, position them as valuable tools for enhancing crop protection and developing environmentally friendly approaches for pest and pathogen control.

PPIs of the PR-6 family can be further classified into subfamilies, including the tomato/potato class II PIs, Bowman–Birk PIs, and Kunitz-type PIs (Datta and Muthukrishnan, 1999; Haq et al., 2004; Christeller and Laing, 2005; Rodríguez-Sifuentes et al., 2020). These subfamilies have been extensively studied in relation to PPIs and AMP research, highlighting their biotechnological applications in this field (Sels et al., 2008; Cotabarren et al., 2020; Godbole and Kharat, 2022). For instance, PPIs like CaCPin-II from *Capsicum annuum* have been found to induce various morphological and physiological alterations in *Candida* species, including pseudohyphae formation, cell swelling, agglutination, growth inhibition, reduced viability, oxidative stress, membrane permeabilization, and metacaspase activation (Cherene et al., 2023). Similarly, inhibitors like the Bowman-Birk-type inhibitor (BBI) from *Oryza sativa* have demonstrated notable antifungal activity against *Pyricularia oryzae* (Qu et al., 2003). Furthermore, trypsin inhibitors from *Psoralea corylifolia* and *Helianthus annuus* have exhibited antifungal effects against *Bactrocera cucurbitae* and *Sclerotinia sclerotiorum* (Giudici et al., 2000; Samiksha et al., 2019). In addition, potato type I inhibitor (StPin1A) from *Solanum tuberosum* and type II (NaPI) from *Nicotiana alata* have been shown to influence the larval growth of *Helicoverpa punctigera* (Dunse et al., 2010). These examples illustrate the diverse and potent effects of PPIs on various biological processes, positioning them as promising candidates for crop protection and formulations against necrotrophs (Nair et al., 2022). Additionally, studies have highlighted PPIs efficacy in managing pests like the castor semi-looper *Achaea janata* and the pod borer *Helicoverpa armigera*, as well as their potential in controlling the growth of gram-positive pathogenic bacteria, including methicillin-sensitive *Staphylococcus aureus* (Gujjarlapudi et al., 2023). Notably, trypsin inhibitor from *Brassica chinense* has demonstrated antibacterial activity against *Pseudomonas aeruginosia* and *Bacillus* species, further underscoring the versatile antimicrobial potential of PPIs (Ngai and Ng, 2004).

Other noteworthy class of PIs discussed in this context is the metalloproteinase inhibitors, also known as carboxypeptidase inhibitors (CPIs). These metalloprotease inhibitors are characterized by their ability to inhibit metallocarboxypeptidases (MCPs) of A and B subfamilies through the interaction of the C-terminal region of CPIs with the catalytic site of carboxypeptidase (Marino-Buslje et al., 2000; Arolas et al., 2005). CPIs contain three conserved intramolecular disulfide bonds, and their presence has been described in a few plant species, primarily within the *Solanaceae* family, including the potato and tomato species (Hass and Ryan, 1981; Lufrano et al., 2015; Molesini et al., 2017; Manara et al., 2020). Notably, metalloproteases, which include the specific targets of CPIs, have been established as key factors in the pathogenesis process of phytopathogenic infections caused by fungi and bacteria (Jashni et al., 2015; Figaj et al., 2019). The knockout of these proteases has shown to significantly impede the ability of pathogens to infiltrate plants (Ökmen et al., 2018; Lelis et al., 2019; Leonard et al., 2020). These findings underscore the potential of MCPs as promising targets for controlling plant pests. In this regard, the biotechnological application of CPIs emerges as a promising avenue in the realm of phytopathogen control, suggesting potential development of innovative strategies to effectively manage phytopathogens.

In plants, the potato CPI is one of the most extensively studied inhibitors within the PPI class. This inhibitor has been associated with various actions in plant defense mechanisms. For instance, research has shown that a potato CPI gene promoted pathogen resistance in transgenic rice (Quilis et al., 2007). In addition, potato CPI has demonstrated antagonist properties against the epidermal growth factor (EGF), displaying antitumor effects. Furthermore, its antifungal activity has been tested, inhibiting in 70% the growth of *Magnaporthe oryzae* and in 40% of *Fusarium verticillioides* in the concentrations of 45 and 40 µM, respectively (Sitjà-Arnau et al., 2005; Quilis et al., 2007; 2014). Despite these advancements, there is still much to explore and understand about CPIs in plants, including their functions and applications in biological processes. Therefore, further investigations are warranted to unravel additional functions and uncover the full biotechnological potential of these peptides, for example, in the development of strategies for crop protection. In this context, the aim of this study is to delve deeper into the CPIs as biotechnological targets, characterizing their class and exploring their antimicrobial potential in *Solanaceae* plants, based on a comprehensive literature review and bioinformatics analyses.

## 2 Materials and methods

### 2.1 Database research

The research for *Solanaceae* carboxypeptidase inhibitors involved a comprehensive search across multiple databases, including UniProt, Protein Data Bank (PDB), KNOTTIN, National Center for Biotechnology Information (NCBI), and MEROPS databases. For UniProt (Bateman et al., 2021), the search terms “metallocarboxypeptidase inhibitor” and “carboxypeptidase inhibitor” were used to identify CPIs. A similar search was conducted in the NCBI database (https://www.ncbi.nlm.nih.gov/). To identify CPI sequences with determined three-dimensional structures, the CPI sequences deposited in UniProt were cross-referenced with the PDB (Berman et al., 2007) ones. The KNOTTIN database (http://knottin.cbs.cnrs.fr) was utilized specifically for selecting CPIs categorized as metallocarboxypeptidase inhibitors. Additionally, the MEROPS database (Rawlings et al., 2018) was consulted to identify CPI sequences belonging to the I37 metalloproteinase inhibitor family. Only sequences confirmed to belong to *Solanaceae* species were selected, while any redundant, incomplete, or uncharacterized sequences were excluded. Sequences with an incomplete cysteine pattern were also excluded from the analyses. This systematic approach enabled the comprehensive identification and selection of relevant CPI sequences, ensuring the inclusion of high-quality, characterized data for further analysis.

### 2.2 Phylogeny and conservation analysis

To investigate the phylogenetic relationships of the selected CPIs from the databases (Supplementary Data S1), a phylogenetic study was performed using the MEGA-X software (Kumar et al., 2018). Sequence alignment was performed using Clustal W. Maximum likelihood phylogeny was carried out using the Jones-Taylor-Thornton (JTT) substitution model and the bootstrap phylogenetic test (Logacheva et al., 2008) with 1,000 replicates. The generated tree was edited using the Interactive Tree of Life (iTOL) tool (Letunic and Bork, 2007). To analyze the conservation patterns of the CPI sequences, Clustal Omega and MView (version 1.63) tools were employed for alignment and subsequent conservation analysis (Madeira et al., 2022).

### 2.3 Physicochemical and antimicrobial potential analysis

The physicochemical properties of CPI sequences were predicted by the software’s ProtParam (Gasteiger et al., 2005) for evaluation of isoelectric point (pI), molecular weight (MW) and charge, and ToxinPred (Gupta et al., 2013) for toxicity. ProtParam utilizes established algorithms to calculate fundamental characteristics such as pI, which is determined based on the distribution of charged residues along the peptide sequence; MW, computed by summing the atomic weights of constituent atoms; and charge, derived from the ionizable groups present in molecules at a given pH. ToxinPred employs machine learning techniques, trained on a diverse dataset of toxic and non-toxic peptides, to predict the potential toxicity of CPI sequences. Assessment of the antimicrobial potential of CPIs by iAMPred (Meher et al., 2017) for prediction of antimicrobial activity (antiviral, antifungal, and antibacterial) and APD3 (Wang et al., 2016) for hydrophobicity and binding potential evaluation. iAMPred employs a machine learning-based approach, trained on features indicative of antimicrobial activity, to predict the peptides’ effectiveness against viruses, fungi, and bacteria. APD3 relies on a combination of physicochemical properties and machine learning models to estimate hydrophobicity, crucial for understanding the peptide’s interactions with aqueous environments, as well as to evaluate binding potential based on Boman Index (Boman, 2003), which involves assessing the peptide’s propensity to form stable interactions with other molecules. Analyzes were performed with CPI sequences that were cut at the third amino acids residue before the first cysteine of the CPI consensus sequence. This pattern was defined by comparing the unknown sequences with the functional sequences of potato and tomato CPIs sequences that originated their three dimensional structures, PDB codes 1H20 and 2HLG respectively. All the bioinformatic analyses relied on predictions generated by those described programs.

### 2.4 Structural feature analysis

The secondary structure standards were predicted using PSIPRED (Buchan and Jones, 2019) software for all selected CPI sequences. PSIPRED employs a machine learning approach, utilizing feedforward neural networks, to predict secondary structure elements such as alpha helices, beta strands and coils, based on position-specific scoring matrices derived from multiple sequence alignments. The PDB structures of CPIs from potato (1H20 code) (González et al., 2003) and tomato (2HLG code) (Uribe et al., 2008) CPIs, determined by Nuclear Magnetic Resonance (NMR) technique, were used to exemplify the structural characteristics of plant CPIs. Furthermore, the PDB structure (4CPA code) of potato CPI interacting with a bovine (*Bos taurus*) metallocarboxypeptidase A (MCPA) (Rees and Lipscomb, 1982), determined by X-ray crystallography, was also used to show and exemplify the main features involved in plant CPIs mechanism of action. The bioinformatic programs PLIP (Adasme et al., 2021) and Dyscovery Studio 2021 (Biovia, 2021) were used to map interactions, considering factors like distance, polarity, and affinity between atoms and/or molecular groups. PLIP employs a combination of geometric and physicochemical criteria, along with sophisticated algorithms, to identify and categorize interactions between ligands and proteins, providing a detailed structural understanding of these interactions. Dyscovery Studio 2021 employs a suite of computational tools and algorithms to analyze molecular interactions, offering insights into binding affinities, thermodynamic parameters, and the energetic landscape of protein-ligand interactions.

### 2.5 Systematic literature search

A systematic literature search was conducted to identify relevant studies pertaining to *Solanaceae* carboxypeptidase inhibitors (CPIs) and their activities against pathogens. The databases Web of Science, Scopus, PubMed, and Embase were extensively searched using the main search terms “metallocarboxypeptidase inhibitor,” “*Solanaceae*,” and “pathogen.” Sub-descriptors of each main term were employed to broaden the search scope. The resulting studies from the four searches were compiled, and any duplicated studies were excluded from the analysis. Furthermore, the reference lists of the identified studies were reviewed to identify additional relevant papers. In the study selection process, certain criteria were applied. Inclusion criteria involved studies focused on “*Solanaceae* carboxypeptidase inhibitor,” “CPI antimicrobial activity,” “CPI resistance action in pathogen infection,” “CPI expression in pathogen infection,” and “CPI inhibition of pathogen metallocarboxypeptidase.” Conversely, exclusion criteria encompassed studies not related to carboxypeptidase inhibitors or their actions in pathogen infection, as well as studies of secondary nature or those investigating other functions of carboxypeptidase. The findings of each included study were carefully tabulated for further analysis. The methodology employed for the literature search adhered to an adapted form of the Preferred Reporting Items for Systematic Reviews and Meta-Analysis (PRISMA) guidelines (Grimshaw et al., 2021). This systematic approach ensured a comprehensive and transparent selection of relevant studies to address the research objectives effectively.

## 3 Results

### 3.1 Characterization of CPI sequences

**FIGURE 1:**
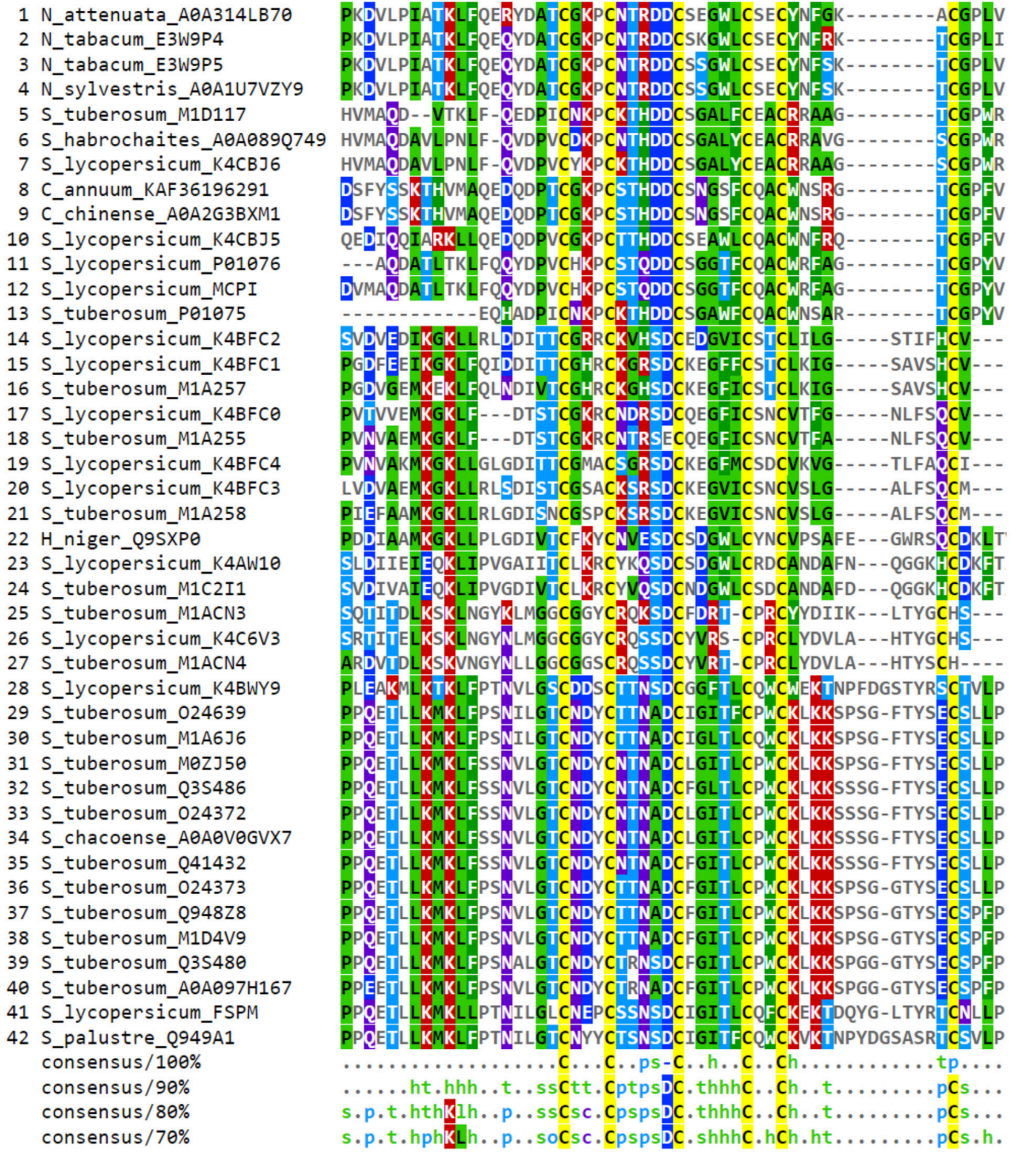
Consensus alignment of *Solanaceae* CPI sequences selected from databases. Global alignment was generated in Clustal Omega and visualized in MView software. The consensus alignment, at various percentages (100%, 90%, 80%, and 70%), provide a clear representation of the shared characteristics among the aligned sequences. Each consensus is accompanied by an MView color map. The color map illustrates the similarity patterns of amino acids at each position in the alignment based on their chemical properties: h (hydrophobic), o (alcohol), p (polar), s (small), and t (turnlike). In instances where an amino acid is identical across all sequences at a particular position, it is indicated by its one-letter code in each consensus. Specific colors highlight amino acids chemical characteristics: cysteines are marked in yellow, hydrophobic amino acids in green, positively charged ones in red, negatively charged in dark blue, and polar amino acids in light blue.

The database search for CPI sequences within the *Solanaceae* plant family yielded a total of 42 deposited sequences, the majority of them were found in more than one database (Supplementary Table S1). These sequences were primarily distributed among the *Solanum*, *Capsicum*, and *Nicotiana* genera, spanning across nine different species. Specifically, the species included *Capsicum annuum*, *Capsicum chinense*, *Hyoscyamus niger*, *Nicotiana attenuata*, *Nicotiana sylvestris*, *Nicotiana tabacum*, *Solanum lycopersicum*, *Solanum palustre*, and *Solanum tuberosum*. Among these sequences, the CPIs from potato and tomato exhibited the highest occurrence, displaying significant sequence variability. Conversely, the carboxypeptidase inhibitors found in *Nicotiana* and *Capsicum* species were less frequently observed and demonstrated greater sequence conservation (Figure 1).

**FIGURE 2:**
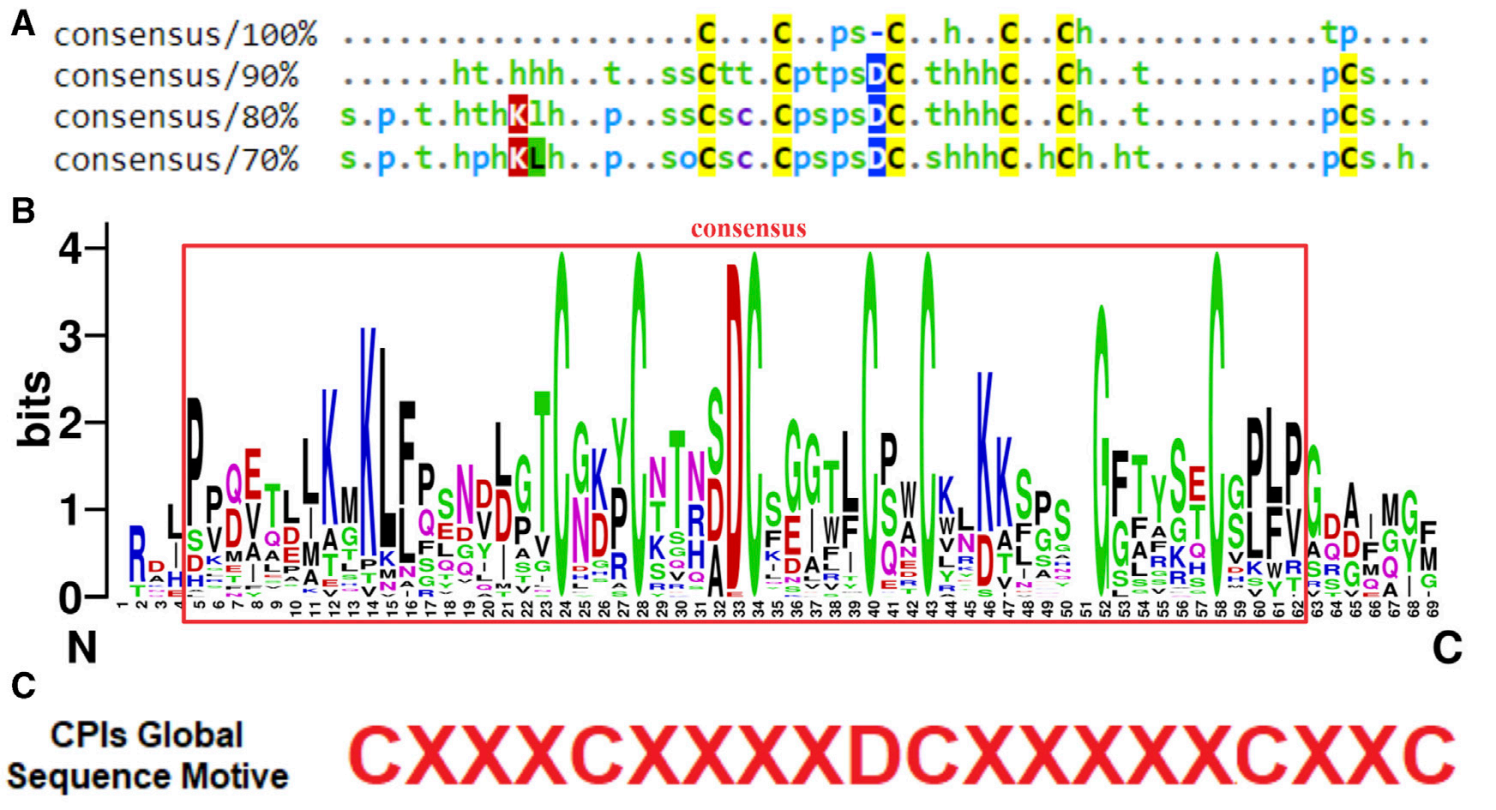
Conservation patterns of *Solanaceae* CPI sequences from databases. **(A)** Consensus standards of aligned sequences generated by MView software showing amino acids similarities. The MView color map classifies amino acids by their chemical characteristics: h (hydrophobic aa); o (alcohol aa); p (polar aa); s (small aa); t (turnlike aa). In instances where an amino acid is identical across all sequences at a particular position, it is indicated by its one-letter code in each consensus. Specific colors highlight amino acids chemical characteristics: cysteines are marked in yellow, hydrophobic amino acids in green, positively charged ones in red, negatively charged in dark blue, and polar amino acids in light blue. **(B)** Logo generated by WebLogo software of *Solanaceae* CPIs multiple alignment. The WebLogo graph indicates the relative frequency of the most common amino acids in each position of the alignment (https://weblogo.berkeley.edu/). Alignment consensus region is marked in red. The numbering of each amino acid position in the alignment is shown on the horizontal axis, where N indicates the N-terminus corresponding to the beginning of the alignment and C the C-terminus corresponding to the end of the alignment. In vertical axis, the bits ranging from 0 to 4 are indicated, which correspond to the frequency of the amino acids in each position. WebLogo color map shows the conserved amino acids colored in different colors according to the classic classification of amino acids by physicochemical characteristics. **(C)** Global characterization motive of *Solanaceae* CPI sequences proposed according to conservation standards of consensus alignment and distances between cysteines.

The conservation patterns related to the characterization of CPI sequences in *Solanaceae* plants are depicted in the consensus alignment of selected sequences obtained from the referred databases (Figure 1). The consensus alignment reveals a high degree of conservation in cysteine residues and in the main regarding their distances between C-C bonds. Specifically, the positions and spacing of the first three cysteine residues are conserved, while the positions and spacing between Cys 4, 5, and 6 exhibit variability. Additionally, the consensus analysis (90%) describes the conservation of amino acid chemical characteristics in the region between Cys I-VI. Notably, different chemical features are observed in the interspace of C-C bonds. For instance, between Cys I-II, turn-like amino acids are prevalent, while polar, small, and turn-like residues dominate between Cys II-III. Hydrophobic residues are mainly found between Cys III-IV, and there is no specific amino acid preference between Cys IV-V. Moreover, the presence of an aspartic acid residue (D) before the third cysteine is conserved in about 90% of the sequences. This alignment position is consistently represented by a negatively charged amino acid in 100% of the aligned sequences. Furthermore, the consensus analyses at 80% and 70% thresholds reveal other interesting patterns, such as the appearance of hydrophobic residues in the carboxy terminal region (C-terminal) (Figures 1, 2A).

The CPIs from the *Solanaceae* family focused in this study exhibited a consensus in terms of amino acid characteristics (Figures 1, 2). The generated logo (Figure 2B) provides a visual representation of the variation and frequency of amino acids at each position, further supporting the consensus of physicochemical features along the CPI sequences. Specially, there is a prominent occurrence of six cysteine residues, as well as an aspartic acid residue at position 33 and hydrophobic amino acids in the C-terminal consensus region. Taking into account the conservation of amino acid standards within the region between C:I-IV, a consensus motif of CXXXCXXXXDCXXXXXCXXC was proposed as a global characterization of CPIs, where “X" represents any amino acid. Remarkably, this consensus motif was observed in 90% of the analyzed CPIs (42 sequences) (Figure 2C).

### 3.2 Distribuction of CPIs

**FIGURE 3:**
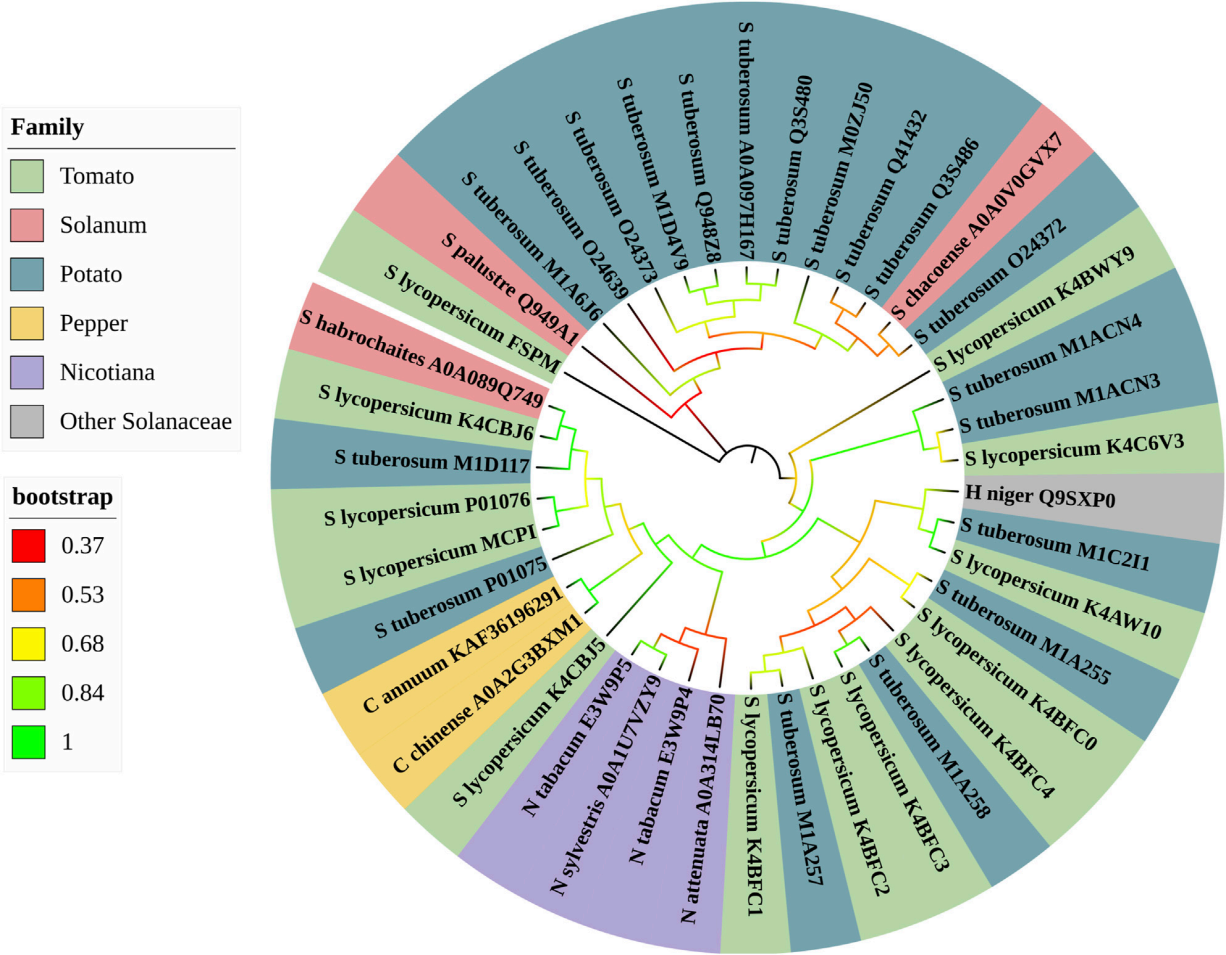
Phylogeny of database *Solanaceae* CPI sequences. Maximum likehood phylogenetic tree was constructed by MEGA X software using the bootstrap phylogenetic test. The tree shows the phylogenetic relationships of *Solanaceae* CPI sequences selected from databases. CPIs are colored according to six families that were classified into *Solanaceae* species and genera. The families are tomato, potato, pepper, *Solanum*, *Nicotiana* and other *Solanaceae*. The Bootstrap variation (0.37-1) is showed in different colors.

The phylogenetic analysis of CPIs from nightshades (Figure 3) revealed their distribution across seven phylogenetic families, based on the species and genera of *Solanaceae* in which CPIs were found. The analysis demonstrated significant bootstrap values (ranging from 0.53 to 1) in the majority of the tree branches, indicating robust support for the phylogenetic relationships. Remarkably, the phylogenetic tree displayed a closer evolutionary proximity and sequence similarity within genera, as evident from the clustering of CPIs from *Capsicum*, *Nicotiana*, and *Solanum* species. In contrast, each clade of the tree contained at least one CPI from the potato and/or tomato families, indicating their evolutionary proximity to the other five solanaceous families. This suggests that potato and tomato CPIs differentiated early in the evolutionary adaptation of carboxypeptidase inhibitor expression in plants, which can be attributed to their higher genetic variability. The presence of these CPIs in multiple clades further underscores their evolutionary significance within the *Solanaceae* family.

### 3.3 Physicochemical standards of CPIs

According to physicochemical prediction analysis, the *Solanaceae* CPIs (Table 1) exhibit a molecular mass ranging between 3.5 and 6.5 kDa. The isoelectric point (pI) of CPIs varies between 4 and 8, influenced by their charge, which varies not only within genera but also between them, spanning from −3 to +3. Specifically, CPIs from *Nicotiana*, *Capsicum*, and *S. habrochaites* are predominantly anionic, while *H. niger* and *S. chacoense* CPIs are neutral. On the other hand, CPIs from *S. tuberosum* and *S. lycopersicum* exhibit a mix of neutral, positive, and negative charges. Most CPIs demonstrate toxicity, although a small subset, comprising three from *S. lycopersicum* and one from *S. tuberosum*, are considered non-toxic. These findings highlight the potential of these peptides as defense agents, given their physicochemical predicted characteristics, which resemble those of antimicrobial peptides and plant pathogenesis related proteins (García-Olmedo et al., 2001; Sels et al., 2008). Notably, the non-toxic CPIs represent interesting targets for the development of biotechnological products, as they pose no harm to humans, are natural molecules, and could be considered eco-friendly or “green” chemicals (no harmful to humans and environment) (Table 1).

**TABLE 1:** Physicochemical properties of *Solanaceae* CPIs.

Species	Code	ProtParam			ToxinPred
		pI^a^	Molecular weight (Da)	Charge	Toxicity
*Capsicum annuum*	KAF36196291	5.30	3,638.97	−1	Toxic
*Capsicum chinense*	A0A2G3BXM1	5.30	3,638.97	−1	Toxic
*Hyoscyamus niger*	Q9SXP0	6.26	6,470.36	0	Toxic
*Nicotiana attenuata*	OIT383771	4.23	4,301.85	−3	Toxic
*Nicotiana sylvestris*	A0A1U7VZY9	4.36	4,337.90	−2	Toxic
*Nicotiana tabacum*	E3W9P4	6.16	4,490.19	0	Toxic
*Nicotiana tabacum*	E3W9P5	4.36	4,337.90	−2	Toxic
*Solanum chacoense*	A0A0V0GVX7	6.08	4,451.11	0	Toxic
*Solanum habrochaites*	A0A089Q749	4.82	4,291.72	−2	Toxic
*Solanum lycopersicum*	K4BWY9	4.04	4,600.05	−3	Toxic
*Solanum lycopersicum*	FSPM	4.78	4,376.00	−1	Toxic
*Solanum lycopersicum*	MCPI	6.78	4,397.01	0	Nontoxic^b^
*Solanum lycopersicum*	K4CBJ6	6.71	4,325.83	0	Toxic
*Solanum lycopersicum*	K4CBJ5	4.66	3,876.36	−2	Nontoxic^b^
*Solanum lycopersicum*	K4BFC2	6.87	3,671.31	0	Toxic
*Solanum lycopersicum*	K4BFC1	8.68	3,635.24	+3	Toxic
*Solanum lycopersicum*	K4BFC0	5.78	3,724.16	0	Toxic
*Solanum lycopersicum*	K4BFC4	6.08	3,566.20	0	Toxic
*Solanum lycopersicum*	K4BFC3	7.77	3,489.06	+1	Nontoxic^b^
*Solanum lycopersicum*	K4AW10	7.79	6,838.50	+1	Toxic
*Solanum lycopersicum*	K4C6V3	7.60	3,849.35	+1	Toxic
*Solanum palustre*	Q949A1	7.76	4,601.21	+1	Toxic
*Solanum tuberosum*	P01075	6.87	4,913.56	0	Toxic
*Solanum tuberosum*	M1A6J5	6.08	4,479.17	0	Toxic
*Solanum tuberosum*	M1D117	7.79	4,357.92	+1	Toxic
*Solanum tuberosum*	M1A257	8.34	3,580.21	+2	Nontoxic^b^
*Solanum tuberosum*	M1A255	7.48	3,738.23	+1	Toxic
*Solanum tuberosum*	M1A258	7.77	3,528.10	+1	Toxic
*Solanum tuberosum*	M1C2I1	5.45	6,058.75	−1	Toxic
*Solanum tuberosum*	M1ACN3	8.27	4,001.59	+2	Toxic
*Solanum tuberosum*	M1ACN4	7.79	3,712.20	+1	Toxic
*Solanum tuberosum*	O24639	6.08	4,482.17	0	Toxic
*Solanum tuberosum*	M1A6J6	6.08	4,479.17	0	Toxic
*Solanum tuberosum*	M0ZJ50	6.08	4,461.15	0	Toxic
*Solanum tuberosum*	Q3S486	6.08	4,485.13	0	Toxic
*Solanum tuberosum*	O24372	6.08	4,451.11	0	Toxic
*Solanum tuberosum*	O24373	6.08	4,392.04	0	Toxic
*Solanum tuberosum*	Q948Z8	6.08	4,410.02	0	Toxic
*Solanum tuberosum*	M1D4V9	6.08	4,410.02	0	Toxic
*Solanum tuberosum*	Q3S480	7.76	4,451.07	+1	Toxic
*Solanum tuberosum*	Q41432	6.08	4,485.13	0	Toxic
*Solanum tuberosum*	A0A097H167	7.76	4,435.07	+1	Toxic

^a^ pI, isoelectric point.

^b^ Nontoxic CPIs are marked in gray color.

### 3.4 Structural features and mechanism of action of CPIs

**FIGURE 4:**
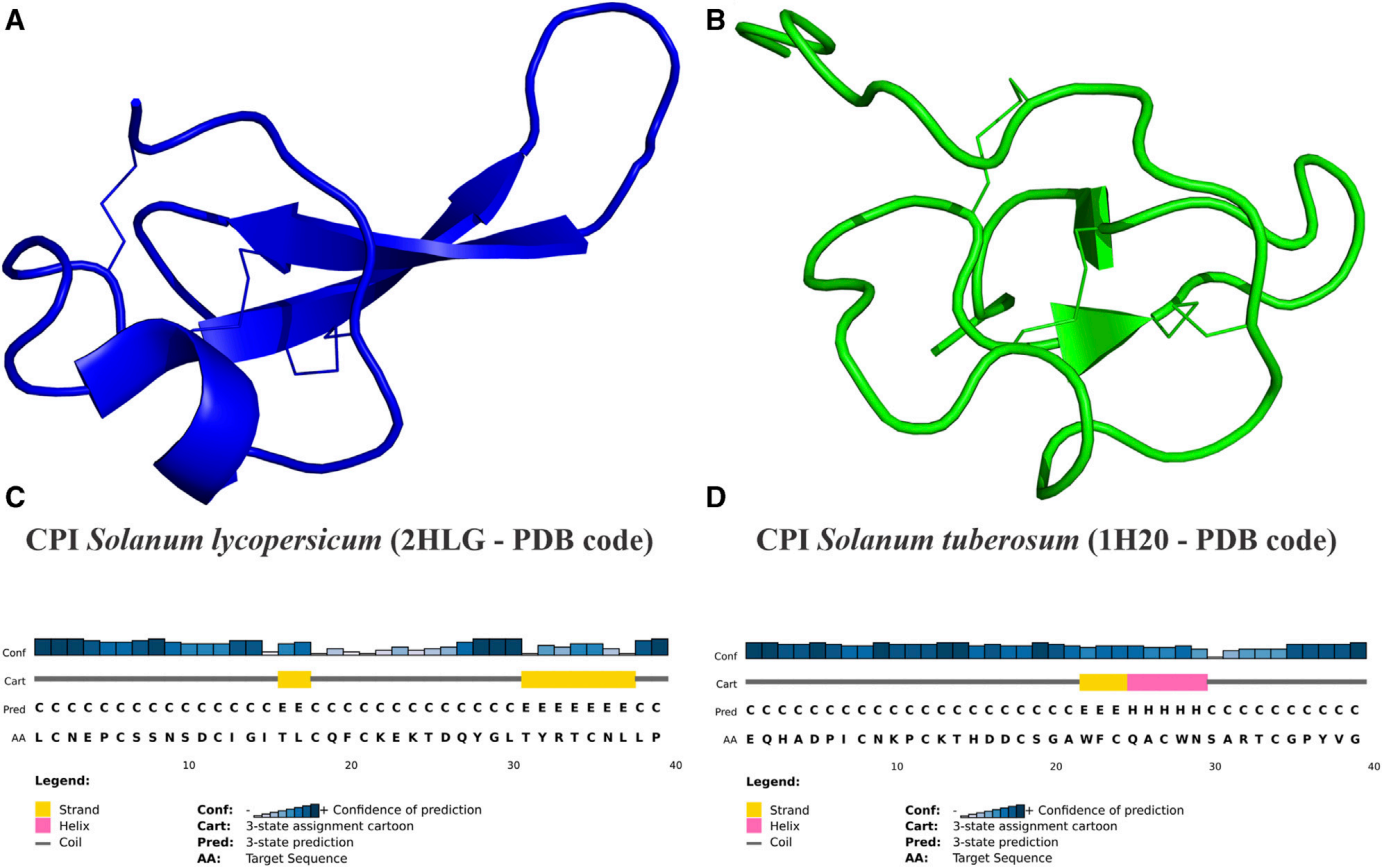
CPIs three-dimensional structures of tomato and potato from PDB database. **(A)** Cartoon representation of tomato (*S. lycopersicum*) CPI structure (PDB code 2HLG) (in blue) showing disulfide binds–I:C2-C19; II:C6-C21; III:C12-C35—(Uribe et al., 2008). **(B)** Cartoon representation of potato (*S. tuberosum*) CPI structure (PDB code 1H20) (in green) showing disulfide bounds–I:C8-C24; II:C12-C27; III:C18-C34—(González et al., 2003). **(C,D)** Sequence secondary patterns of tomato and potato, respectively CPI 3D structure. **(A,B)** The CPIs’ 2HLG and 1H20 structures were determined by NMR (Nuclear Magnetic Resonance) technique. **(C,D)** Sequence secondary patterns of tomato and potato CPIs were predicted by PSIPRED software. Secondary standards of β-strand (E) are represented by yellow bars, α-helix (H) by pink bars and coil (C) by gray bars. Blue graph shows the confidence of standards prediction for each amino acid.

The primary predicted secondary structure patterns observed in *Solanaceae* CPIs involve the presence of β-strands located in the middle of sequences in nearly all analyzed peptides. Additionally, α-helices consistently follow β-sheets, while coil structures are present in all CPIs (Table 2). The secondary predicted structure characteristics and patterns are conserved within the *Nicotiana* and *Capsicum* genera but vary across different genera. Specifically, *Capsicum* CPIs typically contain a single small β-strand in the middle of sequences, while *Nicotiana* CPIs exhibit the same β-strand as *Capsicum*, followed by an α-helix. On the other hand, *Solanum* CPIs show varying secondary structure motifs within the genus and also among different species. Potato and tomato CPIs display a range of secondary structure motifs. In *S. lycopersicum*, sequences generally include at least one β-strand and/or α-helix, following the secondary structure pattern determined by NMR analysis of a tomato CPI (PDB 2HLG–Figures 4A, C) (Uribe et al., 2008). In *S. tuberosum*, CPIs can consist of either a single β-strand or α-helix, or include multiple β-strands and/or α-helices, with some conforming to the secondary structure pattern determined by NMR analysis of a potato CPI (PDB 1H20—Figures 4B, D) (González et al., 2003). CPIs from other *Solanum* species generally follow the pattern characterized for potato or tomato CPIs, while *H. niger* exhibits a distinct secondary structure pattern, with three β-strands along the CPI sequence and a helix at the end (Table 2).

**TABLE 2:** Secondary structures patterns of *Solanaceae* CPIs.

Species	Code	PSIPRED
		Secondary structure patterns^a^
*Capsicum annuum*	KAF36196291	CCCCCCCCCCCCCCCCCEEECCCCCCCCCCCCCCC
*Capsicum chinense*	A0A2G3BXM1	CCCCCCCCCCCCCCCCCEEECCCCCCCCCCCCCCC
*Hyoscyamus niger*	Q9SXP0	CCCCCCCCCCCCCCCCCEEEECCCCCCCCCCCEEECEEEECCCCCHHHHH
*Nicotiana attenuata*	OIT383771	CCCCCCCCCCCCCCCCCEEEHHHCCCCCCCCCCCCCCCCCC
*Nicotiana sylvestris*	A0A1U7VZY9	CCCCCCCCCCCCCCCCCEECHHHHHCCCCCCCCCCCCCCCC
*Nicotiana tabacum*	E3W9P4	CCCCCCCCCCCCCCCCCEEEHHHHHCCCCCCCCCCCCCCCC
*Nicotiana tabacum*	E3W9P5	CCCCCCCCCCCCCCCCCEECHHHHHCCCCCCCCCCCCCCCC
*Solanum chacoense*	A0A0V0GVX7	CCCCCCCCCCCCCCCCCEECCCCCCCCCCCCCCCEEEECCC
*Solanum habrochaites*	A0A089Q749	CCCCCCCCCCCCCCCCCEEEHHHCCCCCCCCCCCCCCCC
*Solanum lycopersicum*	K4BWY9	CCCCCCCCCCCCCCCCCEECCCCCCCCCCCCCCCCCEEECCC
*Solanum lycopersicum*	FSPM	CCCCCCCCCCCCCCCEECCCCCCCCCCCCCEEEEEEECC
*Solanum lycopersicum*	MCPI^b^	CCCCCCCCCCCCCCCCCEEEHHHHCCCCCCCCCCCCCHHCC
*Solanum lycopersicum*	K4CBJ6	CCCCCCCCCCCCCCCCCEEEHHCCCCCCCCCCCCCCCCC
*Solanum lycopersicum*	K4CBJ5^b^	CCCCCCCCCCCCCCCCCEEEHHHHHHCCCCCCCCC
*Solanum lycopersicum*	K4BFC2	CCCCCCCCCCCCCCCCCEECHHCHHHCCCCCCCC
*Solanum lycopersicum*	K4BFC1	CCCCCCCCCCCCCCCCCEEEHHHCCCCCCCCCCC
*Solanum lycopersicum*	K4BFC0	CCCCCCCCCCCCCCCCCEEEHHHCCCCCCCCCCC
*Solanum lycopersicum*	K4BFC4	CCCCCCCCCCCCCCCCCEECHHCCCCCCCCCCCC
*Solanum lycopersicum*	K4BFC3^b^	CCCCCCCCCCCCCCCCCEEEHHCHHCCCCHHHCC
*Solanum lycopersicum*	K4AW10	CCCCCEECCCCCCCCCCEEEECCCCCCCCCCCCEEEECCCCEEEEEEEEE
*Solanum lycopersicum*	K4C6V3	CCCCCCCCCCCCCCCCCCCCCCCCCHHHHCCCCCC
*Solanum palustre*	Q949A1	CCCCCCCCCCCCCCCCCEECCCCCCCCCCCCCCEEEEEEECC
*Solanum tuberosum*	P01075	CCCCCCCCCCCCCCCCCCCCCEEEECCCCCCCCCCCCCCCCCCCCC
*Solanum tuberosum*	M1A6J5	CCCCCCCCCCCCCCCCCEEECCCCCCCCCCCCCCCEEECCC
*Solanum tuberosum*	M1D117	CCCCCCCCCCCCCCCCCEEEHHHCCCCCCCCCCCCCCCC
*Solanum tuberosum*	M1A257^b^	CCCCCCCCCCCCCCCCCEEECCCCCCCCCCCCCC
*Solanum tuberosum*	M1A255	CCCCCCCCCCCCCCCCCEEEHHHCCCHHHHHHCC
*Solanum tuberosum*	M1A258	CCCCCCCCCCCCCCCCCEECHHHHHCCCCCHHCC
*Solanum tuberosum*	M1C2I1	CCCCCCCCCCCCCCCCCEEECCCCCCCCCCCCCCEEEEECCEEEEEEECC
*Solanum tuberosum*	M1ACN3	CCCCCCCCCCCCCCCCCCCCCCCCCHHHCCCCCCC
*Solanum tuberosum*	M1ACN4	CCCCCCCCCCCCCCCCCCCCCCCCCHHHHCCCCC
*Solanum tuberosum*	O24639	CCCCCCCCCCCCCCCCCEECCCCCCCCCCCCCCCCEEEECC
*Solanum tuberosum*	M1A6J6	CCCCCCCCCCCCCCCCCEEECCCCCCCCCCCCCCCEEECCC
*Solanum tuberosum*	M0ZJ50	CCCCCCCCCCCCCCCCCEECCCCCCCCCCCCCCCCEEECCC
*Solanum tuberosum*	Q3S486	CCCCCCCCCCCCCCCCCEECCCCCCCCCCCCCCCEEEECCC
*Solanum tuberosum*	O24372	CCCCCCCCCCCCCCCCCEECCCCCCCCCCCCCCCEEEECCC
*Solanum tuberosum*	O24373	CCCCCCCCCCCCCCCCCEECCHHCCCCCCCCCCEEEEEECC
*Solanum tuberosum*	Q948Z8	CCCCCCCCCCCCCCCCCEECCCCCCCCCCCCCCCCCCEECC
*Solanum tuberosum*	M1D4V9	CCCCCCCCCCCCCCCCCEECCCCCCCCCCCCCCCCCCEECC
*Solanum tuberosum*	Q3S480	CCCCCCCCCCCCCCCCCEECCCCCCCCCCCCCCCCCCEEC
*Solanum tuberosum*	Q41432	CCCCCCCCCCCCCCCCCEECCHHCCCCCCCCCCCEEEECCC
*Solanum tuberosum*	A0A097H167	CCCCCCCCCCCCCCCCCEECCCCCCCCCCCCCCCCCCEECC

^a^ H, E, and C represent respectively α-helix, β-strand and coil structure patterns.

^b^ Nontoxic CPIs are marked in gray color.

The mechanism of action of plant CPIs, specifically the potato CPI (PCI), has been elucidated through studies conducted on bovine (*Bos taurus*) metallocarboxypeptidase A (MCPA) (Rees and Lipscomb, 1982) and more recently on *Aedes aegypti* metallocarboxypeptidase B (MCPB) (Gavor et al., 2021). The catalytic zinc-binding site of metalloproteases is formed through the interaction of three nitrogen atoms from histidine residues and an oxygen atom from glutamate or aspartate. Within this site, specific residues are essential for catalysis and substrate binding, and they are grouped into five subsites: S1’, S1, S2, S3, and S4. The S1’ subsite, composed of Asn144, Arg145, Tyr248, and Xaa255, is highly conserved among the M14 family of metalloproteases and plays a crucial role in fixing and neutralizing the carboxyl group of the substrate. The S1, S2, S3, and S4 subsites, involving residues such as Arg127, Leu/Ile247, Glu270, Arg71, Asp142, Ser197, Tyr198, Ser199, Phe279, Glu122, Arg124, and Lys128, contribute to substrate binding and catalysis (Auld, 2013).

**FIGURE 5:**
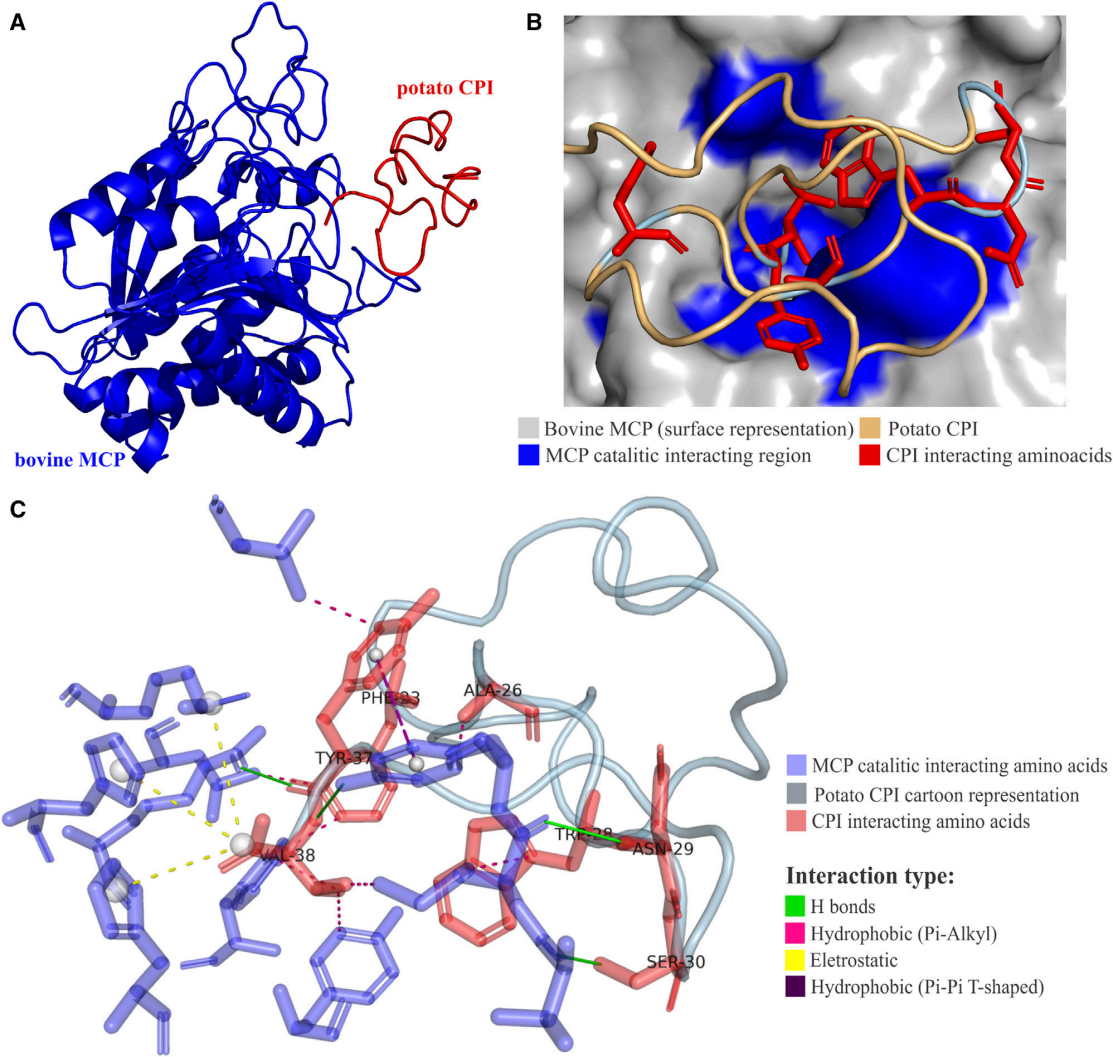
Potato CPI mechanism of action. **(A)** Three-dimensional structure of potato CPI complex with bovine (*Bos taurus*) metallocarboxypeptidase A (MCPA) (PDB code 4CPA). CPI is shown in red and MCPA in blue. **(B)** Interaction anchoring region of potato CPI in bovine MCPA. CPA catalytic region is destacted in blue and CPI interacting amino acids in red. **(C)** Interactions between catalytic site of MCPA and amino acids of potato CPI generated by PLIP software. The H bonds type interactions are shown in green, hydrophobic π-alkyl type in pink, hydrophobic π-π T-shaped type in purple and electrostatic in yellow. Amino acids of CPA catalytic site are destacted in blue and CPI interacting amino acids in red. The 4CPA PDB structure was determined by X-ray crystallography technique (Rees and Lipscomb, 1982).

The interaction between PCI and bovine MCPA reveals a complex network of interactions. The C-terminal amino acids of PCI engage in multiple interactions with the catalytic site amino acids of MCPA (Marino-Buslje et al., 2000) (Figures 5A, B). These interactions include electrostatic, hydrogen bond, hydrophobic π-π T-shaped, and T π-alkyl interactions. Notably, the C-terminal residues of PCI, such as V38, form interactions with MCPA residues in subsites S1’, S1, and S2. Additionally, interactions involving PCI residues Y37, N29, and A26 with MCPA subsites S1’, S1, and S2 contribute to the stability and specificity of the complex (Figure 5C; Table 3). The C-terminal PCI residues implicated in the interaction maintain a hydrophobic characteristic that is consistently observed across the analyzed group of CPIs sequences. In the consensus alignment, the last four amino acid residues share a hydrophobic nature in each position for nearly all CPI sequences. These characteristics strongly suggest that hydrophobic amino acids play a pivotal role in the mechanism of inhibition for plant CPIs, along with predominantly electrostatic and hydrophobic interactions. Understanding these intrinsic interactions between potato CPI and metalloproteases provides valuable insights into the inhibition mechanism and the precise binding modes involved. This knowledge enhances the understanding of how plant CPIs effectively inhibit the activity of target metalloproteases and guides the design of more potent and specific inhibitors. Such insights hold promise for the development of novel therapeutic strategies targeting metalloprotease-related diseases and pathogen infections.

**TABLE 3:** Mainly interactions involved in potato CPI inhibition mechanism in bovine MCP.

Interaction	Distance (Å)	Types	From	To
MCP:ARG127:NH1 - CPI:VAL38:OXT	4.27	Electrostatic - Attractive Charge	Positive	Negative
MCP:ARG127:NH2 - CPI:VAL38:O	5.42	Electrostatic - Attractive Charge	Positive	Negative
MCP:ARG145:NH1 - CPI:VAL38:OXT	4.70	Electrostatic - Attractive Charge	Positive	Negative
MCP:ARG71:NH1 - CPI:TYR37:O	2.87	Conventional Hydrogen Bond	H-Donor	H-Acceptor
MCP:TYR248:OH - CPI:VAL38:OXT	2.95	Conventional Hydrogen Bond	H-Donor	H-Acceptor
CPI:ASN29:N - MCP:ILE247:O	2.85	Conventional Hydrogen Bond	H-Donor	H-Acceptor
CPI:VAL38:N - MCP:TYR248:OH	3.02	Conventional Hydrogen Bond	H-Donor	H-Acceptor
MCP:TYR248 - CPI:TYR37	5.09	Hydrophobic - Pi-Pi T-shaped	Pi-Orbitals	Pi-Orbitals
MCP:TYR198 - CPI:VAL38	5.39	Hydrophobic - Pi-Alkyl	Pi-Orbitals	Alkyl
MCP:TYR248 - CPI:ALA26	4.25	Hydrophobic - Pi-Alkyl	Pi-Orbitals	Alkyl

### 3.5 Potential of CPIs as antimicrobial and defense agents

The bioinformatics analysis of *Solanaceae* CPIs has revealed their significant antimicrobial potential. Most CPIs were predicted to exhibit strong antimicrobial activity, particularly against bacteria, while the predicted values for antiviral and antifungal activity were relatively lower. The analysis of CPIs in the AMP database (APD3) showed that a majority of the analyzed CPIs had high predicted hydrophobicity ratios and demonstrated binding potential for antimicrobial action (Table 4). The Boman Index, a measure of protein-binding potential, showed positive values for CPIs, indicating a high likelihood of antimicrobial activity (Boman, 2003). These CPIs possess partial hydrophobicity and exhibit cationic or anionic properties, which further enhance their potential as defense agents and AMPs. Notably, the non-toxic CPIs from *S. lycopersicum* codes MCPI, K4CBJ5 and K4BFC3, and from *S. tuberosum* code M1A257 showed promising characteristics, making them attractive targets for studies regarding the development of antimicrobial agents.

**TABLE 4:** Antimicrobial potential of *Solanaceae* CPIs.

Species	Code	iAMPred - antimicrobial action^a^			APD3 - AMP prediction	
		Antibacterial	Antiviral	Antifungal	Hidrofobicity (%/score)	Binding potential
*Capsicum annuum*	KAF36196291	0.97	0.64	0.84	31%/4.11	1.85 kcal/mol
*Capsicum chinense*	A0A2G3BXM1	0.97	0.64	0.84	31%/4.11	1.85 kcal/mol
*Hyoscyamus niger*	Q9SXP0	0.85	0.80	0.48	42%/2.33	0.96 kcal/mol
*Nicotiana attenuata*	OIT383771	0.87	0.69	0.67	41%/7.34	1.21 kcal/mol
*Nicotiana sylvestris*	A0A1U7VZY9	0.88	0.48	0.79	39%/5.62	1.40 kcal/mol
*Nicotiana tabacum*	E3W9P4	0.95	0.72	0.91	39%/6.68	1.66 kcal/mol
*Nicotiana tabacum*	E3W9P5	0.88	0.48	0.79	39%/5.62	1.40 kcal/mol
*Solanum chacoense*	A0A0V0GVX7	0.98	0.91	0.96	39%/1.05	0.88 kcal/mol
*Solanum habrochaites*	A0A089Q749	0.53	0.27	0.47	36%/9.98	2.60 kcal/mol
*Solanum lycopersicum*	K4BWY9	0.94	0.83	0.83	33%/2.64	1.73 kcal/mol
*Solanum lycopersicum*	FSPM	0.89	0.76	0.86	36%/5.71	1.58 kcal/mol
*Solanum lycopersicum*	MCPI^b^	0.81	0.38	0.79	43%/3.58	1.04 kcal/mol
*Solanum lycopersicum*	K4CBJ6	0.71	0.28	0.86	36%/8.48	2.41 kcal/mol
*Solanum lycopersicum*	K4CBJ5^b^	0.75	0.69	0.50	43%/3.71	1.40 kcal/mol
*Solanum lycopersicum*	K4BFC2	0.93	0.54	0.65	47%/3.69	1.00 kcal/mol
*Solanum lycopersicum*	K4BFC1	0.99	0.84	0.98	41%/4.59	1.48 kcal/mol
*Solanum lycopersicum*	K4BFC0	0.98	0.83	0.94	38%/4.92	2.19 kcal/mol
*Solanum lycopersicum*	K4BFC4	0.97	0.67	0.96	50%/3.84	0.73 kcal/mol
*Solanum lycopersicum*	K4BFC3^b^	0.95	0.75	0.94	47%/4.06	0.96 kcal/mol
*Solanum lycopersicum*	K4AW10	0.99	0.84	0.96	37%/3.67	1.97 kcal/mol
*Solanum lycopersicum*	K4C6V3	0.91	0.71	0.95	34%/0.73	1.69 kcal/mol
*Solanum palustre*	Q949A1	0.99	0.91	0.96	36%/0.70	1.28 kcal/mol
*Solanum tuberosum*	P01075	0.89	0.35	0.84	43%/3.81	1.11 kcal/mol
*Solanum tuberosum*	M1A6J5	0.97	0.86	0.96	39%/1.22	0.86 kcal/mol
*Solanum tuberosum*	M1D117	0.69	0.30	0.78	38%/9.96	2.75 kcal/mol
*Solanum tuberosum*	M1A257^b^	0.99	0.86	0.96	44%/4.70	0.93 kcal/mol
*Solanum tuberosum*	M1A255	0.98	0.72	0.92	41%/4.78	1.93 kcal/mol
*Solanum tuberosum*	M1A258	0.96	0.82	0.89	44%/4.62	1.14 kcal/mol
*Solanum tuberosum*	M1C2I1	0.91	0.65	0.81	40%/6.81	1.87 kcal/mol
*Solanum tuberosum*	M1ACN3	0.96	0.81	0.98	31%/2.15	2.06 kcal/mol
*Solanum tuberosum*	M1ACN4	0.95	0.92	0.96	35%/1.47	1.76 kcal/mol
*Solanum tuberosum*	O24639	0.99	0.94	0.98	39%/0.77	0.75 kcal/mol
*Solanum tuberosum*	M1A6J6	0.97	0.86	0.96	39%/1.22	0.86 kcal/mol
*Solanum tuberosum*	M0ZJ50	0.99	0.92	0.97	39%/1.37	0.80 kcal/mol
*Solanum tuberosum*	Q3S486	0.98	0.84	0.96	39%/0.23	0.93 kcal/mol
*Solanum tuberosum*	O24372	0.98	0.91	0.96	39%/1.05	0.88 kcal/mol
*Solanum tuberosum*	O24373	0.99	0.90	0.97	40%/0.60	0.90 kcal/mol
*Solanum tuberosum*	Q948Z8	0.99	0.90	0.98	34%/2.10	0.96 kcal/mol
*Solanum tuberosum*	M1D4V9	0.99	0.90	0.98	34%/2.10	0.96 kcal/mol
*Solanum tuberosum*	Q3S480	0.99	0.89	0.97	32%/2.61	1.29 kcal/mol
*Solanum tuberosum*	Q41432	0.99	0.91	0.97	39%/0.48	0.93 kcal/mol
*Solanum tuberosum*	A0A097H167	0.99	0.92	0.99	34%/2.65	1.16 kcal/mol

^a^ The values of each type of antimicrobial action represents the probability between 0 and 1 of the sequences having antibacterial, antiviral, and/or antifungal action where 1 is the maximum probability.

^b^ Nontoxic CPIs are marked in gray color.

**FIGURE 6:**
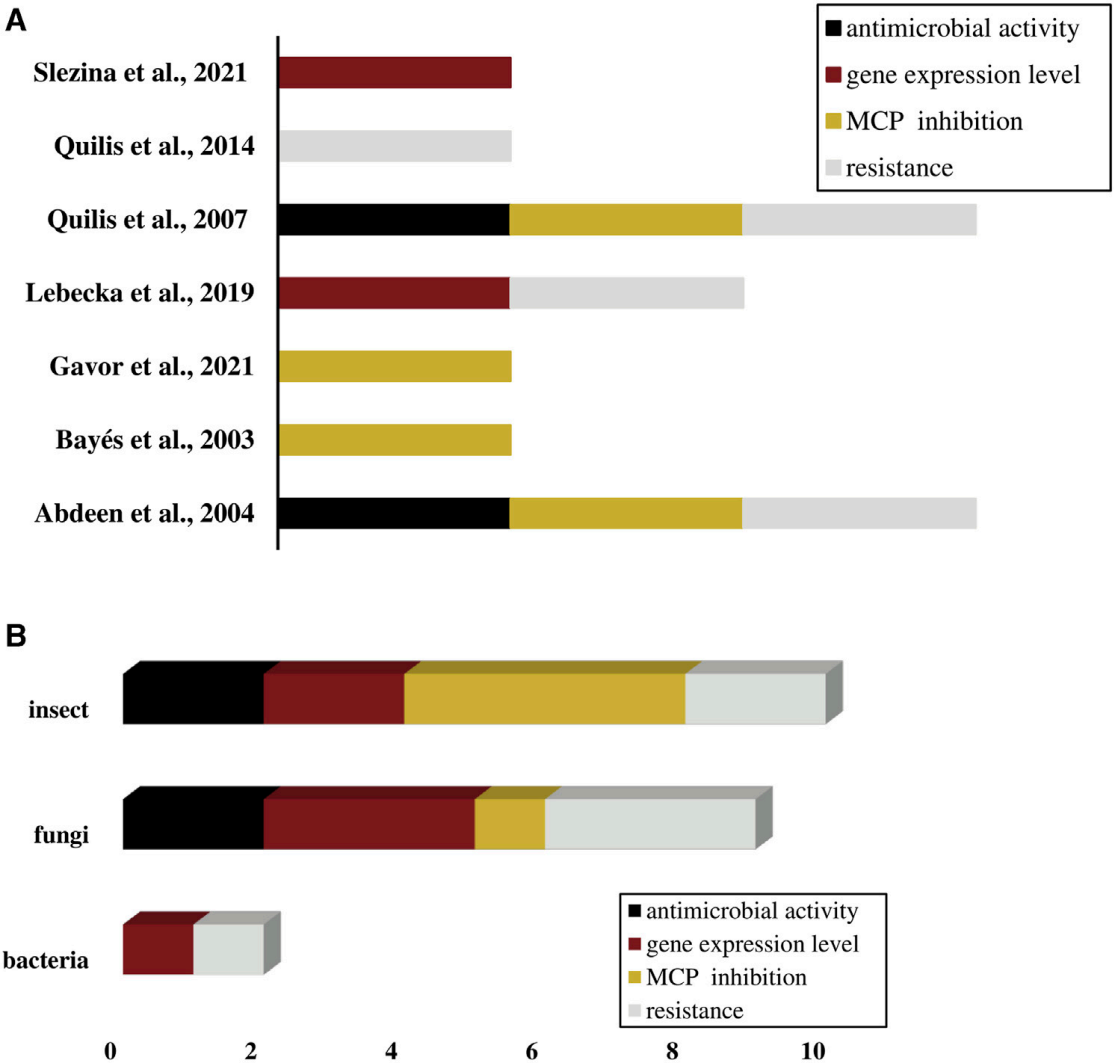
CPIs activities against pathogens described in studies (*n* = 7). **(A)** CPIs activities identified per study. **(B)** CPIs activities frequency per type of pathogen. **(A,B)** Activities are shown in different colors: antimicrobial activity in black, gene expression level in wine color, MCP inhibition in dark yellow and resistance in light gray.

The systematic review search identified seven studies that described the activities of *Solanaceae* CPIs against pathogens. Detailed information regarding the search strategy were provided in the Supplementary Figure S1; Supplementary Table S2. The activities identified in the studies encompassed the inhibition of pathogen MCPs, resistance mechanisms, CPI expression during pathogen infection, and antimicrobial effects. Some studies reported multiple types of CPI activities against different pathogens, indicating the versatility of CPIs in counter various pathogens. The predominant focus of CPI actions was directed towards fungi and insects, encompassing the four main CPI activities (Figures 6A, B). However, three significant research gaps need to be addressed in the field of CPIs’ actions against pathogens. Firstly, there is a limited number of studies available, indicating the need for more research in this area. Secondly, the existing studies tend to concentrate primarily on fungi and insects, leaving other types of pathogens underrepresented, and third, the studies concentrate the rearches just in the potato and tomato CPIs. It is essential to encourage and support further research on CPI activities against a broader range of pathogens, including bacteria, viruses, and other types of microbes, and also to investigate CPIs from other *Solanaceae* species and their actions against pathogens.

The main finds of CPIs activities in studies were (Supplementary Table S3): 1) potato CPI antimicrobial activities were related to the inhibition growth of *Fusarium verticillioides* and *Magnaporthe oryzae* fungi (Quilis et al., 2007) and the mortality of *Heliothis* obsolete insect larvae (Abdeen et al., 2005); 2) resistance potato CPI induction reduced syntoms of diseases caused by the fungus *Fusarium verticillioides* and *Magnaporthe oryzae* (Quilis et al., 2007; 2014), by the insects *Heliothis obsolete*, *Liriomyza trifolii*, and *Chilo suppressalis* (Abdeen et al., 2005; Quilis et al., 2014), and by the bacteria *Dickeya solani* (Lebecka et al., 2019); 3) the expression of two tomato peptides which shares identity with tomato CPI were regulated in response to *Fusarium oxysporum* and *Fusarium sambucinum* fungal infestation (Slezina et al., 2021). The potato CPI inhibition against pathogens MCPs were one of the major finding results, the peptide was able to inhibit A/B MCPs of the pathogens *Aedes aegypti*, *Magnaporthe oryzae*, *Helicoverpa armigera*, and *Liriomyza trifolii* in very low concentrations raging between 0.7 and 25 µM (Bayés et al., 2003; Abdeen et al., 2005; Quilis et al., 2007; Gavor et al., 2021) (Table 5), which shows one more time the CPI potential to be used in development of biotechnological products for pathogen control and plant defense.

**TABLE 5:** Potato (PCI) inhibition of pathogen MCPs evaluated in studies (*n* = 4).

Pathogen	PCI^a^ (µM)	MCP inhibition (%)
*Aedes aegypti*	0.7	100
*Magnaporthe oryzae*	2.5	98
*Helicoverpa armigera*	5.0	100
*Liriomyza trifolii*	25.0	75

^a^ PCI, potato carboxypeptidase inhibitor.

## 4 Discussion

The characterization of CPI sequences within the *Solanaceae* plant family provided valuable insights about their distribution, sequence variability, conservation patterns, and a newly proposed consensus motif in this work. The CPIs were primarily identified in the *Solanum*, *Capsicum*, and *Nicotiana* genera, spanning across nine different species. Notably, potato and tomato CPI sequences exhibited the highest occurrence and showed significant sequence variability. The plants and culture of *Solanum lycopersicum* and *Solanum tuberosum*, along with other species from the *Solanum*, *Capsicum*, and *Nicotiana* genera, have been extensively studied due to their agricultural and/or nutritional importance, as well as their suitability as model organisms (Kimura and Sinha, 2008; Pombo et al., 2020; Bánfalvi et al., 2021). These plants are of significant interest in scientific research and breeding programs due to their economic value, widespread cultivation, and diverse applications in the food industry (Añibarro-Ortega et al., 2022). The identification of CPI sequences primarily in the *Solanum*, *Capsicum*, and *Nicotiana* genera aligns with the focus on these agriculturally important species. The extensive study of these genera and species provides a solid foundation for investigating the distribution, functional roles, and potential applications of CPIs within the *Solanaceae* family. These plants play a crucial role in AMP research, with protease inhibitors (PIs) in *Solanaceae* species having been characterized for their antimicrobial activity against insects, bacteria, and fungi. Additionally, *Solanaceae* plants are known for their resistance properties, with some species in the *Solanum* genus having more than 400 identified resistance genes (Yang et al., 2014; Nair et al., 2022). Studying CPIs, particularly within this family, could pave the way for utilizing CPI-mediated resistance in genetic enhancement strategies for vegetable crops, targeting resistance against pathogens, for example,

The consensus alignment of selected CPI sequences revealed a remarkable conservation of cysteine residues and their distances between C-C bonds. The chemical characteristics of the amino acid residues also exhibited sequence conservation within the region between Cys I-VI, with distinct features in the sequences present in the interspaces between each two cysteines. Furthermore, the presence of an aspartic acid residue (D) at a consistent position was detected. Similar conservation patterns to that observed in the CPI sequences, including cysteine distance patterns and position conservation, are found in pathogens related proteins and antimicrobial peptides such as cysteine-rich peptides and defensin peptides (Dias and Franco, 2015; Slezina et al., 2021; Ma et al., 2023). These peptides are known for their role in defense mechanisms and their contribution to the formation of highly stable peptide structures. Based on the observed conservation patterns, a consensus motif of CXXXCXXXXDCXXXXXCXXC was proposed as a global characterization of CPIs within the *Solanaceae* family for the first time. It is important to note that this motif suggests the presence of specific features for CPIs, as they show distinct characteristics that differentiate them from other protease inhibitors and cysteine rich peptides. This work provides a clearer and more concise presentation of the CPI sequences’ characterization within the *Solanaceae* family, emphasizing their distribution, sequence variability, conservation patterns, and the proposed consensus motif. This motif characterization could potentially catalyze new searches for the discovery and study of additional CPIs within family genomes, for example. Cysteine-rich peptides have been scrutinized for their cysteine patterns through whole-genome transcriptome sequencing of *Solanum lycopersicum* (Slezina et al., 2021). Distinct classes of AMPs, such as thionins, defensins, and heveins, have been defined by their specific motifs based on cysteine patterns (Slezina and Odintsova, 2023). In light of the proposed consensus motif for CPIs, this class could also be characterized by its unique sequence attributes.

The phylogeny shed light on the evolutionary relationships and diversification of CPIs within the *Solanaceae* family. The significant bootstrap values indicate that the clustering and grouping of CPIs in the phylogenetic tree are reliable indicators of their evolutionary relatedness. The close evolutionary proximity and sequence similarity observed within the *Capsicum*, *Nicotiana*, and *Solanum* genera support the idea of a common ancestry and recent diversification within these genera. This is further supported by the sequence similarities observed within *Capsicum* and *Nicotiana* species, reinforcing their evolutionary relationships. The presence of CPIs from the potato and tomato families in multiple clades of the tree suggests that these CPIs differentiated early in the evolutionary adaptation of carboxypeptidase inhibitor expression in plants. The higher genetic variability of potato and tomato may have contributed to their distinct evolutionary path within the *Solanaceae* family. The significance of potato and tomato CPIs is further emphasized by their presence across different clades, highlighting their evolutionary importance within family. A previous study (Manara et al., 2020) constructed a phylogenetic tree involving *N. tabacum*, *S. lycopersicum*, and *S. tuberosum* CPIs, demonstrating that the more similar the CPIs are, the closer their evolutionary proximity. Another study, which characterized a new CPI named β-lybatide from *Lycium barbarum*, also constructed a phylogenetic tree of protease inhibitors. The resulting clade corresponding to plant CPIs exhibited similar relationships as described in this study, wherein sequences from the same genus showed greater conservation and closer phylogenetic ties (Huang et al., 2021). This finding further supports the evolutionary relationships described in this study, providing additional evidence for the relatedness and diversification of CPIs within the *Solanaceae* family.

The predicted physicochemical characteristics of *Solanaceae* CPIs shed light on their properties as defense agents. These CPIs typically have a molecular mass ranging between 3.5 and 6.5 kDa, exhibit variations in charge (ranging from cationic to anionic), and many of them show toxicity. These characteristics indicate their potential role in plant defense mechanisms. Interestingly, the tomato CPI has already been implicated in responding to abiotic stress and shows increased expression levels after pathogen infection, suggesting its role as defense agent (Slezina et al., 2021; Guan et al., 2022). It is worth noting that these features are similar to antimicrobial peptides and plant pathogenesis related proteins, which are known to be involved in plant defense.

In the realm of pathogen control, non-toxic CPIs emerge as compelling subjects for biotechnological applications. They serve as natural compounds that pose no harm to humans or the environment, aligning with the ethos of eco-friendly, “green” chemical products. The quest for stable molecules and metabolites sourced from nature is gaining momentum, particularly in the context of developing environmentally-conscious biopesticides (Nair et al., 2022; Zhang et al., 2023). PIs play a pivotal role in safeguarding plants against invading pathogens by disrupting their physiological processes (Godbole and Kharat, 2022). Understanding the physicochemical stability of PIs, especially concerning temperature and pH, is crucial for their effective deployment in agronomic settings. Plant PIs (PPIs), distinguished by a high cysteine content and the formation of robust disulfide bridges, exhibit varying degrees of stability (Nair et al., 2022). Some retain their activity even under elevated temperatures, exemplified by the trypsin inhibitor’s resilience (Dokka and Davuluri, 2014). Given the central role of PIs in plant defense, it is imperative to scrutinize their stability within specific plant families. In this context, the study of CPIs within the *Solanaceae* family stands out as a promising avenue for investigation, owing to their distinctive sequence and physicochemical characteristics reveled in this study.

The secondary predicted standards of *Solanaceae* CPIs varies in the number of β-strands and/or α-helices along the sequences. CPIs structural features follow in some parts the elucidated NMR tridimensional structures of potato (1H20—PDB code) (González et al., 2003) and tomato (2HLG–PDB code), as the presence of at least one β-strand in the sequence middle, which suggest that the deposited sequences can act similarly of them. The recently discovered *Solanaceae* CPI, β-lybatide from *Lycium barbarum*, also corroborates the β-strand patterns predicted for CPI sequences (Huang et al., 2021). In addition, some sequences follow secondary patterns of β-strands and/or α-helices appearance of other CPIs, as the tick *Rhipicephalus bursa* TCI (Pantoja-Uceda et al., 2008), which possess two β-strands and one α-helice, and the leech *Hirudo medicinalis* LCI (Reverter et al., 2000) that possess three β-strands and one α-helice. These structural characteristics, along with the high sequence diversity, indicate the potential broad spectrum of action for CPIs. The presence of multiple gene copies and isoforms suggests that these CPIs can act more rapidly and efficiently, potentially resulting in a more potent defense response.

The mechanism of action of *S. tuberosum* PCI involves the multiple binding interactions with the S1′, S1 and S2 catalytic subsites of *B. taurus* MCPA and englobes the hydrogen bond, electrostatic and hydrophobic interactions which form a highly stable and strong interaction of C-terminal PCI region and MCPA (Rees and Lipscomb, 1982). Recently, the PCI mechanism of inhibition in *Aedes aegypti* metallocarboxypeptidase B (MCPB) was elucidated and is similar to the MCPA inhibition. PCI interacts with *A. aegypti* MPCB by their C terminal (V38, Y37, S30, N29, and G25) residues, anchoring in the catalytic site of CPB that comprise S1’, S1, S2 and S3 subsites. The bind potency was almost the same as MCPA bind (Gavor et al., 2021). The *R. bursa* TCI and *Ascaris* ACI also have similar mechanisms of action for MCPs A and B (Arolas et al., 2005; Sanglas et al., 2009). These comparisons show the ability of PCI inhibits two types of MCPs and the conservation of CPI mechanism of action despite the differences among kingdoms, what could be used to develop products with CPIs that target to inhibit carboxypeptidases, which act as pathogenic virulence factors in host infection.

The hydrophobic amino acid characteristics in the C-terminal region are shared among CPIs elucidated mechanisms. The conservation of these hydrophobic features in the final four amino acid residues across the majority of CPI sequences, as observed in the consensus alignment, underscores their critical role in the inhibition mechanism of both plant and animal CPIs. These interactions, combined with predominantly electrostatic and hydrophobic interactions, collectively contribute to the conservation of action mechanisms among CPIs across diverse domain. These finds offer a wealth of practical applications. These range from designing drugs targeting carboxypeptidases for treating infections to developing eco-friendly pest control agents. In agriculture, CPIs could lead to the creation of disease-resistant crops, increasing yields. Moreover, potential therapeutic interventions in human health and advancements in enzyme engineering for bioprocessing are on the horizon. CPIs also serve as valuable research tools and have diagnostic applications. Furthermore, they may enhance the production of biopharmaceuticals (Nair et al., 2022). This body of knowledge opens avenues for transformative impacts on pharmaceuticals, agriculture, and biotechnology, with ongoing research likely uncovering further opportunities.

The *Solanaceae* CPIs demonstrate a significant antimicrobial potential, as evidenced by several characteristics. Firstly, they exhibit negative values for Boman Index binding potential, indicating a propensity to interact with microbial targets. Secondly, their hydrophobicity ratios fall within the range of 30%–50%, suggesting a favorable environment for antimicrobial activity. Furthermore, the majority of CPIs show high antimicrobial activity prediction scores ranging between 0.80 and 0.99, further supporting their effectiveness against microbial pathogens, and exhibit variations in charge. Lastly, their toxicity levels contribute to their potential as defense agents. These combined attributes, including Boman Index binding potential, hydrophobicity ratios, antimicrobial activity prediction scores, charge variations, and toxicity indicate the strong antimicrobial potential of *Solanaceae* CPIs. The main findings of literature systematic review about the activities of *Solanaceae* CPIs against pathogens corroborates the CPIs antimicrobial potential. The activities are discussed below.

The systematic review focusing on solanaceous CPIs selected 7 studies that, in general, demonstrate the applicability of ICPs. These studies have investigated the effectiveness of CPIs combating different types of pathogens, including insects, bacteria, and fungi. CPIs in the studies acted through four types of activities, which were MCPs inhibition, inducible resistance, gene expression during pathogen infection and antimicrobial activities. In *Solanum tuberosum*, PCI has shown significant resistance against insects such as *Heliothis obsoleta* and *Liriomyza trifolii*. Transgenic tomato lines expressing PCI exhibited high levels of resistance, resulting in larval death, reduced weight, and inhibition of larval development in *Heliothis obsoleta*. PCI also effectively inhibited the carboxypeptidase activity of *Liriomyza trifolii* (Abdeen et al., 2005). Similarly, PCI displayed inhibition against *Helicoverpa armigera* and *Aedes aegypti*, showing its potential as an effective insecticide (Bayés et al., 2003; Gavor et al., 2021). Moreover, studies have demonstrated the involvement of CPIs in plant defense against bacterial and fungal infections. The potato MCPI exhibited significant expression in response to *Dickeya solani* infection, indicating its defensive role against bacterial pathogens (Lebecka et al., 2019). Additionally, PCI demonstrated antifungal activity against *Fusarium verticillioides* and *Magnaporthe oryzae*. Transgenic rice plants expressing the PCI gene exhibited enhanced resistance, resulting in reduced fungal growth and disease symptoms (Quilis et al., 2007; 2014). In *Solanum lycopersicum* (tomato), knottin-like peptides known as SlKnot1 and SlKnot2, which share sequence identity with metallocarboxypeptidase inhibitors, were found to be involved in the plant defense against *Fusarium oxysporum* and *Fusarium sambucinum fungi*. The expression of these peptides was regulated in response to fungal infestation, suggesting their role in plant protection (Slezina et al., 2021).

In summary, our study provides a thorough characterization of *Solanaceae* CPI sequences, revealing insights into their distribution, sequence variability, conservation patterns, and proposing a consensus motif (CXXXCXXXXDCXXXXXCXXC). These findings align with those observed in pathogen-related proteins, highlighting their potential as potent defense agents. The phylogenetic analysis illuminates the evolutionary relationships and diversification within the *Solanaceae* family, particularly in the *Capsicum*, *Nicotiana*, and *Solanum* genera. The physicochemical characteristics of *Solanaceae* CPIs, including molecular mass, charge variation, and toxicity, underscore their potential as formidable defense agents, bearing similarities to antimicrobial peptides. Moreover, the secondary structures of these peptides, exhibiting variations in the number of β-strands and α-helices, suggest a broad spectrum of action. The CPI mechanism of inhibition, involving multiple binding interactions with carboxypeptidase targets, further underscores their potential as potent antimicrobial agents. Studies have demonstrated the multifaceted activities of *Solanaceae* CPIs against various pathogens, including insects, bacteria, and fungi, paving the way for their application in biotechnology, including the development of disease-resistant crops and eco-friendly pest control methods. Looking ahead, future research could delve deeper into specific applications in agriculture, biotechnology, and pharmaceuticals, exploring the mechanisms of action and stability of *Solanaceae* CPIs. Additionally, the environmental benefits of employing non-toxic CPIs as defense agents align perfectly with the global shift towards sustainable and eco-friendly agricultural practices. The potential to enhance resistance against pathogens in vegetable crops, particularly in economically vital genera like *Solanum*, *Capsicum*, and *Nicotiana*, holds great promise for future agricultural practices. Moreover, the implications of *Solanaceae* CPIs in human health cannot be overlooked, with applications in developing novel therapeutic interventions or improving biopharmaceutical production offering exciting prospects for the pharmaceutical industry. Lastly, the proposal to establish a new subclass for *Solanaceae*-derived carboxypeptidase inhibitors is a significant step towards focused research efforts, promising to deepen our understanding of these unique protease inhibitors and catalyze advancements in plant defense mechanisms. Overall, this comprehensive study unveils the remarkable potential of *Solanaceae* CPIs as versatile defense agents with applications spanning agriculture, biotechnology, and pharmaceuticals, suggesting transformative impacts on multiple industries, from pharmaceuticals to agriculture, and beyond.

## Data Availability

The original contributions presented in the study are included in the article/Supplementary Material, further inquiries can be directed to the corresponding author.
