# Supplementary material for: Carboxypeptidase inhibitors from Solanaceae as a new subclass of pathogenesis related peptide aiming biotechnological targets for plant defense
Source: Front Mol Biosci. 2023 Nov 16;10:1259026. doi: 10.3389/fmolb.2023.1259026 (PMC10687636; doi:10.3389/fmolb.2023.1259026)
Supplement: Supplementary file 1 [file Table1.pdf]

Supplementary Table 1 – Cross-reference of CPI sequences from *Solanaceae* species among databases

**Species**	**Codes of CPI sequences in each database**				
	**UNIPROT**	**KNOTTIN**	**PDB**	**MEROPS**	**NCBI**
Capsicum annuum					KAF36196291
Capsicum chinense	A0A2G3BXM1				PHU112411 / TYK035941
Hyoscyamus niger		Q9SXP0			
*Lycium barbarum**				MER1384952	
Nicotiana attenuata	A0A314LB70				OIT383771
Nicotiana sylvestris	A0A1U7VZY9			MER0638028	
Nicotiana tabacum	E3W9P4	E3W9P4		MER0233254	BAJ257811
Nicotiana tabacum	E3W9P5 / A0A1S4BQ42	E3W9P5 / A0A1S4BQ42			BAJ257821
Solanum chacoense	A0A0V0GVX7	A0A0V0GVX7			
Solanum habrochaites	A0A089Q749	A0A089Q749			
Solanum lycopersicum		K4BWY9			
Solanum lycopersicum	P14903	FSPM	2HLG	MER0621328	NP0012347621
Solanum lycopersicum	P01076	MCPI		MER0018229	CAA419731 / NP0012339341
Solanum lycopersicum		K4CBJ6		MER0413511	XP0042427161
Solanum lycopersicum		K4CBJ5		MER0413510	
Solanum lycopersicum		K4BFC2			
Solanum lycopersicum		K4BFC1			
Solanum lycopersicum		K4BFC0			
Solanum lycopersicum		K4BFC4			
Solanum lycopersicum		K4BFC3			
Solanum lycopersicum		K4AW10			
Solanum lycopersicum		K4C6V3			
Solanum palustre	Q949A1	Q949A1			CAC481961
*Solanum pennellii**					XP0150807211
Solanum tuberosum	P01075 / Q9SBH8	MCPI	1H20	MER0018230	NP0012750481 / AAC951301
Solanum tuberosum	M1A6J5				

Solanum tuberosum	M1D117	M1D117		
Solanum tuberosum		M1A257		
Solanum tuberosum		M1A255		
Solanum tuberosum		M1A258		
Solanum tuberosum		M1C2I1		
Solanum tuberosum		M1ACN3		
Solanum tuberosum		M1ACN4		
Solanum tuberosum		O24639		BAA214961 / BAA215001 / BAA214971 / NP0012753751 / AAZ941831
Solanum tuberosum		M1A6J6		
Solanum tuberosum		M0ZJ50		
Solanum tuberosum		Q3S486		NP0012754211 / NP0012751501 / AAZ941961 / AAZ941881 / AAZ941781
Solanum tuberosum	O24372	O24372		AIT422221 / BAA214931
Solanum tuberosum	Q948Z8	O24373		BAA214951
Solanum tuberosum	M1D4V9 / A0A097H170	Q948Z8		CAC482271
Solanum tuberosum	Q3S480	M1D4V9		AIT422191 / AIT422181
Solanum tuberosum	Q41432	Q3S480		NP0012754881 / AAZ941941 / AAZ941801
Solanum tuberosum	A0A097H167	Q41432		AIT422231 / AIT422211 / AIT422201 / BAA214941 / AAC496011
Solanum tuberosum		A0A097H167		AIT422171
*Solanum tuberosum**			MER0615019	
*Solanum tuberosum**	A0A0A0VBW2			

*Fasta sequences of *Solanaceae* carboxypeptidase inhibitors found in databases, but not included in analyses – colored by light gray.
