# Supplementary material for: Carboxypeptidase inhibitors from Solanaceae as a new subclass of pathogenesis related peptide aiming biotechnological targets for plant defense
Source: Front Mol Biosci. 2023 Nov 16;10:1259026. doi: 10.3389/fmolb.2023.1259026 (PMC10687636; doi:10.3389/fmolb.2023.1259026)
Supplement: Supplementary file 2 [file Table2.pdf]

Supplementary Table 2 – Detailed search strategy with search filters and number of studies recovered in electronic databases

PubMed-MEDLINE – Search filters	Records
**#1 Metallocarboxypeptidase inhibitor:** (“metallocarboxypeptidase inhibitor*”[Title/Abstract]) OR (“metalloproteinase inhibitor*”[Title/Abstract]) OR (“carboxypeptidase inhibitor*”[Title/Abstract])	2,488
**#2 *Solanaceae*:** *(Solanaceae*[MeSH Terms]) OR (potato[Title/Abstract]) OR (tomato[Title/Abstract]) OR (*Solanum**[Title/Abstract]) OR (*Capsicum**[Title/Abstract]) OR (*Nicotiana**[Title/Abstract]) OR (*Datura**[Title/Abstract]) OR (*Hyoscyamus**[Title/Abstract]) OR (tobacco[Title/Abstract]) OR (pepper[Title/Abstract])	210,654
**#3 Pathogen:** (pathogen*[Title/Abstract]) OR (phytopathogen*[Title/Abstract]) OR ("plant pathogen*"[Title/Abstract])	1,112,620
**#4 Combined search:** #1 AND #2 AND #3	9
**SCOPUS – Search filters**	**Records**
**#1 Metallocarboxypeptidase inhibitor:** TITLE-ABS-KEY ("metallocarboxypeptidase inhibitor*" OR "metalloproteinase inhibitor*" OR "carboxypeptidase inhibitor*")	134,089
**#2 *Solanaceae*:** TITLE-ABS-KEY (*Solanaceae* OR potato OR tomato OR *Solanum** OR *Capsicum** OR *Nicotiana** OR *Datura** OR *Hyoscyamus** OR tobacco OR pepper)	450,354
**#3 Pathogen:** TITLE-ABS-KEY (pathogen* OR phytopathogen* OR "plant pathogen*")	1,640,381
**#4 Combined search:** #1 AND #2 AND #3	45
**Web of Science – Search filters**	**Records**
**#1 Metallocarboxypeptidase inhibitor:** ((TS=(("metallocarboxypeptidase inhibitor*")) OR TS=("metalloproteinase inhibitor*")) OR TS=("carboxypeptidase inhibitor*"))	4,498
**#2 *Solanaceae*:** (((((((((TS=(*Solanaceae*)) OR TS=(potato)) OR TS=(tomato)) OR TS=(“*Solanum**”)) OR TS=(“*Capsicum**”)) OR TS=(“*Nicotiana**”)) OR TS=(“*Datura**”)) OR TS=(“*Hyoscyamus**”)) OR TS=(tobacco)) OR TS=(pepper))	406,500
**#3 Pathogen:** (((TS=(“pathogen*”)) OR TS=(“phytopathogen*”)) OR TS=("plant pathogen*"))	1,180,123
**#4 Combined search:** #1 AND #2 AND #3	12
**EMBASE – Search filters**	**Records**
**#1 Metallocarboxypeptidase inhibitor:** 'metallocarboxypeptidase inhibitor*':ti,ab,kw OR 'metalloproteinase inhibitor*':ti,ab,kw OR 'carboxypeptidase inhibitor*':ti,ab,kw	3,007

**#2 *Solanaceae:*** *Solanaceae*:ti,ab,kw OR potato:ti,ab,kw OR tomato:ti,ab,kw OR '*Solanum**':ti,ab,kw OR '*Capsicum**':ti,ab,kw OR '*Nicotiana**':ti,ab,kw OR '*Datura**':ti,ab,kw OR '*Hyoscyamus**':ti,ab,kw OR tobacco:ti,ab,kw OR pepper:ti,ab,kw OR *Solanaceae*/de	242,752
**#3 Pathogen:** 'pathogen*':ti,ab,kw OR 'phytopathogen*':ti,ab,kw OR 'plant pathogen':ti,ab,kw OR 'phytopathogen'/de	1,389,574
**#4 Combined search:** #1 AND #2 AND #3	8

Database searches were finalized on July 01st, 2023 at 19:37 pm.
