# Supplementary material for: Carboxypeptidase inhibitors from Solanaceae as a new subclass of pathogenesis related peptide aiming biotechnological targets for plant defense
Source: Front Mol Biosci. 2023 Nov 16;10:1259026. doi: 10.3389/fmolb.2023.1259026 (PMC10687636; doi:10.3389/fmolb.2023.1259026)
Supplement: Supplementary file 3 [file Table3.pdf]

**Supplementary Table 2** – Main findings of studies (n=7) showing *Solanaceae* CPIs activities against pathogens

| Studies | CPI | Species | Pathogen | Main Findings |
|---|---|---|---|---|
| Abdeen et al., 2005 | PCI | *Solanum tuberosum* (potato) | *Heliothis obsoleta* (insect) *Liriomyza trifolii* (insect) | • Tomato homozygous transgenic lines expressing PCI presented high levels of resistance against *H. obsoleta* and *L. trifolii*;<br>• In *H. obsoleta* homo transgenic PCI lines caused 30.4% larvae death, reduced larval weight and consumed leaf area in 40% and inhibit the development stage of larvae fed in more than 70%;<br>• PCI inhibited nearly 75% of *L. trifolii* carboxypeptidase catalytic function in a concentration of 25 µM;<br>• In *L. trifolii* the resistance was similar to that in *H. obsoleta*. |
| Bayés et al., 2003 | PCI | *Solanum tuberosum* (potato) | *Helicoverpa armigera* (insect) | • PCI inhibited 100% the metallocarboxypeptidase CPAHa activity of *H. armigera* in concentration of 5µM;<br>• PCI presented $K_i$ value of 65 pM (competitive inhibition) displaying highly sensitive inhibition in a $10^{-12}$ concentration ratio. |
| Gavor et al., 2021 | PCI | *Solanum tuberosum* (potato) | *Aedes aegypti* (insect) | • PCI inhibited 100% the metallocarboxypeptidase CPBAe1 catalytic activity of *A. aegypti* in concentration of 697 nM;<br>• In inhibition PCI presented $K_i$ value of 14.70 nM (competitive inhibition) and $IC_{50}$ of 25 nM;<br>• PCI displayed highly sensitive in the inhibition of the insect CPB in a $10^{-9}$ concentration ratio. |
| Lebecka et al., 2019 | MCPI | *Solanum tuberosum* (potato) | *Dickeya solani* (bacteria) | • Potato MCPI exhibited significant expression (274.4-fold) in potato diploid resistant cultivar DG 00–270 after *D. solani* infection;<br>• MCPI could contributes to resistance of cultivar DG 00–270 due its higher abundance in this cultivar than in DG 08–305, indicating the defensive role of these proteins in the response to bacterial inoculation. |

| | | | | |
|---|---|---|---|---|
| Quilis et al., 2007 | PCI | *Solanum tuberosum* (potato) | *Chilo suppressalis* (insect)<br>*Fusarium verticillioides* (fungi)<br>*Magnaporthe oryzae* (fungi) | • PCI antifungal activity inhibited in 70% the growth of *M. oryzae* and in 40% of *F. verticillioides* in the concentrations of 45 µM and 40µM, respectively;<br>• *M. oryzae* cultures in the presence of PCI revealed severe changes in fungal morphology, such as hyphal shortening and abnormal bed-like structures of hyphae;<br>• Transgenic rice plants expressing the PCI gene display enhanced resistance in *M. oryzae* and *F. verticillioides* inhibiting the fungal growth and so causing syntoms diminution of the blast and bakanae diseases of rice, respectively;<br>• PCI inhibits M. oryzae CPB in 98% by the concentration of 2.5 µM;<br>• Transgenic rice lines expressing PCI decreased in 50% the level of CPB activity present in the gut extracts of *C. suppressalis*. |
| Quilis et al., 2014 | MPI-PCI | *Solanum tuberosum* (potato) | *Chilo suppressalis* (insect)<br>*Magnaporthe oryzae* (fungi) | • Transgenic rice expressing hibrid mpi-pci gene revealed a significant reduction between 64.6%–39.6% in weight gain in the *C. suppressalis* larvae fed on this rice of and also in their development;<br>• Transgenic rice expressing mpi-pci showed reduction in infestation by *C. suppressalis*;<br>• MPI-CPI transcript accumulation in leaves indicates that mpi promoter drive high level of transgene expression in *C. suppressalis* infested rice plants;<br>• MPI-PCI Transgenic rice reduced in 50% the formation of empty panicles, a condition of *C. suppressalis* disease;<br>• Expression of MPI-PCI in rice drives resistance against *M. oryzae* showing reduction in disease syntoms as small lesions and diminution of leaves affected area. |
| Slezina et al., 2021 | SlKnot1<br>SlKnot2 | *Solanum lycopersicum* (tomato) | *Fusarium oxysporum* (fungi)<br>*Fusarium sambucinum* (fungi) | • SlKnot1 and SlKnot2 knottin like peptides showed 100% sequence identity to the metallo- carboxypeptidase inhibitors of tomato;<br>• *F. oxysporum* infestation in tomato plants up-regulate the expression of SlKnot2 gene and suppress SlKnot1;<br>• *F. sambucinum* infestation in pretreated plants up-regulate the expression of both 1 and 2 SlKnot genes, more significantly it enhanced expression of the SlKnot2 gene. |
