# Supplementary material for: Carboxypeptidase inhibitors from Solanaceae as a new subclass of pathogenesis related peptide aiming biotechnological targets for plant defense
Source: Front Mol Biosci. 2023 Nov 16;10:1259026. doi: 10.3389/fmolb.2023.1259026 (PMC10687636; doi:10.3389/fmolb.2023.1259026)
Supplement: Supplementary file 6 [file Image1.pdf]

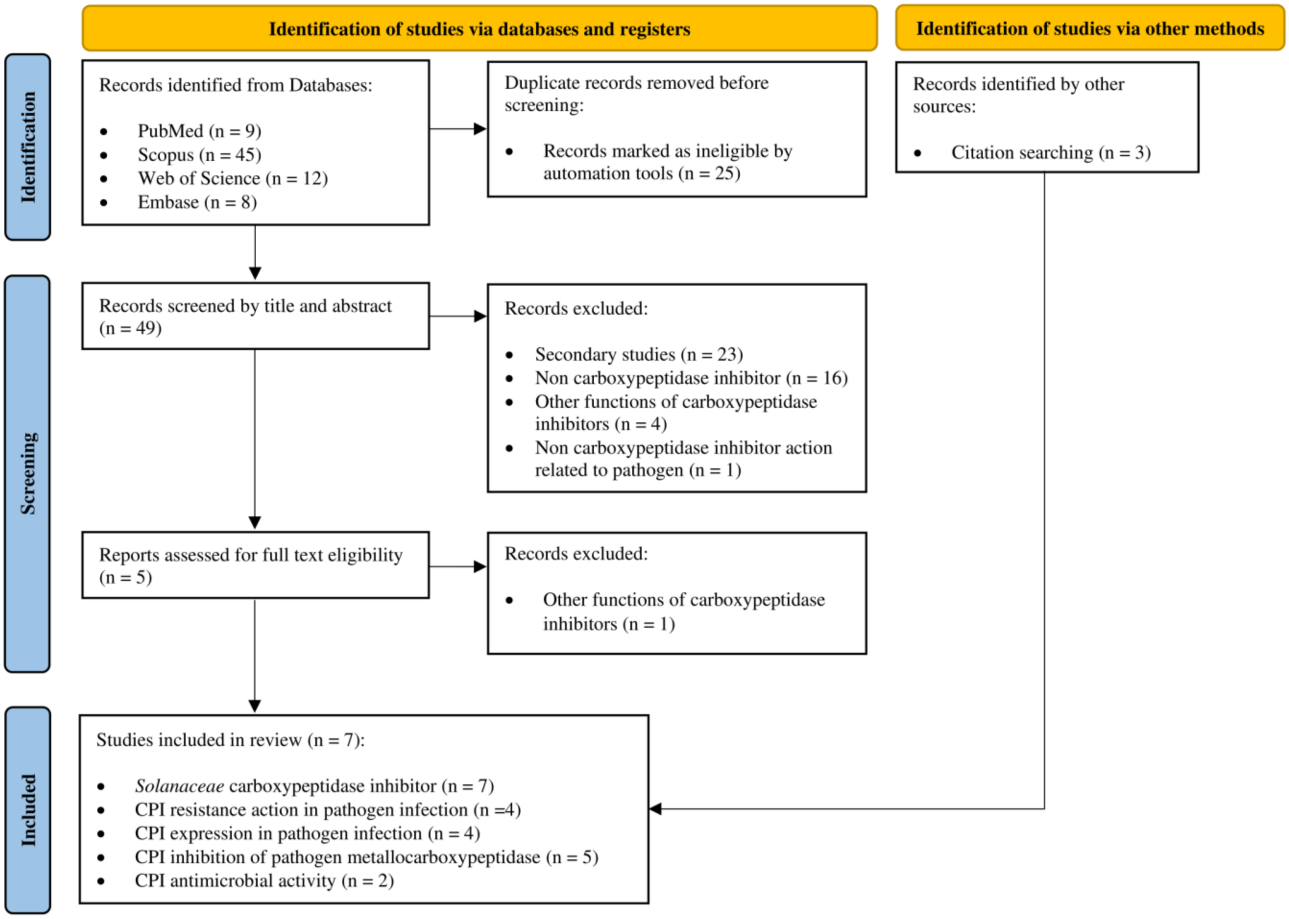

Supplementary Figure S1 – Flow diagram of bibliographic search strategy of studies related to *Solanaceae* CPIs activities against pathogens. The methodology followed an adaptation of PRISMA guidelines (Grimshaw et al., 2021).
